# Supplementary material for: Prospective contributions of biomass pyrolysis to China’s 2050 carbon reduction and renewable energy goals
Source: Nat Commun. 2021 Mar 16;12:1698. doi: 10.1038/s41467-021-21868-z (PMC7966788; doi:10.1038/s41467-021-21868-z)
Supplement: Supplementary file 1 — Supplementary Information [file 41467_2021_21868_MOESM1_ESM.pdf]

Supplementary Information for

Prospective contributions of biomass pyrolysis to China's 2050 carbon reduction and renewable energy goals

Qing Yang[1,2,3,4]*, Hewen Zhou[1,3], , Pietro Bartocci[5], Francesco Fantozzi[5], Ondřej Mašek[6], Foster A. Agblevor[7], Zhiyu Wei[3,4], Haiping Yang[1,3,4], Hanping Chen[1,3,4]*, Xi Lu[8], Guoqian Chen[9], Chuguang Zheng [1], Chris P. Nielsen[2], Michael B. McElroy[2]*

[*] Corresponding author. E-mail: qyang@hust.edu.cn, hp.chen@163.com, mbm@seas.harvard.edu;

Supplementary Note 1: Model simplification for Aspen Plus simulation

Considering the difference between the actual pyrolysis process and the simulation calculation, corresponding simplifications and assumptions need to be made when modeling with the Aspen Plus simulator.

In the Aspen Plus simulator, the process is also divided into two separate processes. First, the biomass is decomposed into elemental components in the RStoic reactor, according to the elemental analysis. Then, it is mixed with oxygen in the RGibbs equilibrium reactor module to carry out the relevant gas phase reaction and produce high-temperature flue gas.

These are the following assumptions adopted in the model: 1) the ash in biomass is defined as an inert component, whose catalytic effect is ignored during pyrolysis; 2) the secondary reactions of volatiles are ignored in the middle-low temperature pyrolysis process; 3) the reaction process is in steady state and its reaction parameters do not change over time; and 4) the temperature of the biomass in the reactor is uniform, ignoring the temperature gradient in the radial direction.

Supplementary Note 2: Model development for Aspen Plus simulation

The pyrolysis process includes six sub-processes: pretreatment, intermediate pyrolysis, condensation, gas storage and supply, power generation and combustion processes. Every process is described in detailed below, and the Aspen Plus unit operation block description is listed in Supplementary Table 1.

Pretreatment section

The agricultural residue stream is fed into a multiple roll crusher (CRUSH) in which the feedstock size is reduced to less than 5 cm in diameter. Then, the feedstock goes through the biomass dryer (DRYER) at an operating temperature of 150 °C to reduce the water content, vaporizing the water bound in the feedstock. The energy (QSUPP2) required for drying is supplied by the flue gas (DRY-AIR) from the first-stage separator (FIR-EXCH) of hot vapor. The feedstock then goes into the intermediate pyrolysis section.

Intermediate pyrolysis section

Two model blocks (PYR-EDCO, PYRO-REC) are implemented to simulate the process of intermediate pyrolysis. In the yield reactor (PYR-EDCO), the biomass is thermally decomposed into subcomponents (cellulose, hemicellulose, lignin and extractives), which then pass into the moving-bed reactor block (PYRO-REC). As it is shown in Supplementary Fig. 1, the biochar (HOT-

CHAR) leaves the reactor at the bottom, and the hot vapor (HOTVAPOR) is released from the top. In the simulation model, the rate-based chemical reactions of each biomass subcomponent is launched in the RCSTR block implemented with the multistep reaction kinetics of biomass pyrolysis developed by Ranzi et al.[1]. Considering the situation of back-mixing in the pyrolysis process, the reactor is simulated with the RCSTR block in series, and each chemical reaction is assigned to one block. When carrying out the process simulation, the yield distribution of biochar, bio-oil and pyrolysis gas can be adjusted by changing the resident time slightly.

Condensation section

There are two major vapor-liquid separators in the condenser section, which is composed of the HeatX block and the Flash2 block in the simulation. In the first-stage, which is implemented to separate the heavy condensable gases in the hot vapor, the outlet temperature of the hot stream is set at 150 °C. The hot vapor from the pyrolysis reactor is cooled down using the cold flue after heat exchange with pyrolysis reactor. The components of the second-stage separator in the simulation are the same as those contained the first-stage one, while the outlet temperature of the hot stream is set at 50 °C. The vapor from the first separator is cooled down using cold water in a heat exchanger. Following the light condensation separator comes the purification tower (SPR-SEP), which can help to remove the tar and the acid components from the pyrolysis gas using recycled water.

Gas storage and supply section

There are two gas tanks to store the pyrolysis gas in the demonstration plant. Nevertheless, there is no need to implement a block for gas storage in the simulation. Thus, in this section, only the gas splitter (GAS-SPI) is set to allocate the pyrolysis gas based on the practical demand. It is assumed that 20% of the total pyrolysis gas produced is used for household heating to replace direct combustion of crop residues now used instead for pyrolysis, and the remainder is used as fuel for power generation.

Power generation section

Given to the higher efficiency for small power generations within the gas turbine, the gas turbines are adopted as generator sets in power generation system. In the simulation process, the 80% pyrolysis gas (20% for household heating) would be mixed with the heated air, and then combusted in the Gibbs equilibrium reactor (BOILER). Thus, the high-temperature flue gas (HOT-

FUME) from the combustion reactor can be used in the power generation to get electricity.

Combustion section

The combustion section is simulated with a yield reactor (FUEL-DEC) and a Gibbs reactor (FUEL-COM). In the Aspen simulator, the Gibbs reactor calculates the multi-phase chemical equilibrium by minimizing the Gibbs free energy[2]. Unreacted biomass goes into the yield reactor where it is decomposed into its constituent elements, based on the biomass ultimate analysis. Next, mixed with the heated air, the biomass fuel (same as biomass feedstock) combusts in the Gibbs reactor, which can generate the high temperature flue gases that can be used to supply the heat for pyrolysis reactions. The air supplied by the fuel combustion is heated by the hot stream (HOT-AIR4), which is used to cool down the hot biochar in the prior step, shown in Supplementary Fig. 1

Supplementary Table 1: Aspen Plus unit operation block description

Block name (Aspen block)	Block parameters	Description
DRYER (Ryield)	Pressure=1 atm; T=150 ℃	Separates the water in conventional components
PYR-DECO ((Ryield)	Pressure=1 atm; T=250 ℃	Separates the subcomponents (including cellulose, hemicellulose, lignin and extractives)
FIR-EXCH (HeatX)	Hot stream outlet temperature=150 ℃	Cooling down the hot vapor from pyrolysis reactor by the cold flue
FIR-SEP (Flash2)	Pressure=3 bar; T=150 ℃	Separate the heavy condensable (such as Levoglucosan and Free fatty acids) in hot vapor
SEC-EXCH (HeatX)	Hot stream outlet temperature=50 ℃	Cooling down the vapor from first separator by the cold water
SEC-SEP (Flash2)	Pressure=3 bar; T=50 ℃	Separate the light material (such as Glycol-aldehyde and CH_3OH)
CYC-MIX (Mixer)	Pressure=1 bar	Mix the crude pyrolysis gas with separated stream (mainly recycling water) from SPR-SEP
CYC-PUMP (Pump)	Discharge pressure=0.5 bar	Pump separated stream from SPR-SEP
SPR-SEP (Flash2)	Pressure=3 bar; T=25 ℃	Separate the tar and acid of the pyrolysis gas
Boiler (RGibbs)	Pressure=0.8 atm	Pyrolysis gas combustion process
Turb (Compr)	Discharge pressure=0.15 bar	Turbine steam expansion
AIR-PRH2 (HeatX)	Hot stream outlet temperature=100 ℃	Air preheating by fume from gas turbine
COOL-AIR (Compr)	Discharge pressure=0.8 atm	Air compression

AIR-COOL (HeatX)	Hot stream outlet temperature=150 ℃	Biochar cooling by cooled air
WAT-COOL (HeatX)	Hot stream outlet temperature=100 ℃	Biochar cooling by cooled water
AIR-BURN (Compr)	Discharge pressure=0.8 atm	Air compression
AIR-PRH (HeatX)	Hot stream outlet temperature=50 ℃	Air preheating by hot fume heated by biochar
FUEL-DEC (RStoic)	Pressure=1 atm; T=25 ℃	Separate the constituent elements based on the biomass ultimate analysis
FUEL-COM (RGibbs)	Pressure=1 atm	Biomass fuel combustion process
HEATSUPP (Heater)	Pressure=1 atm	Heat exchange providing heat to pyrolysis reactor
HEAT-WST (Heater)	Pressure=1 atm	Heat exchange providing heat to biomass dryer
**Materials and stream flows for 1 t rapeseed biomass feedstock input at 650 ℃**		
S-FUEL	Value=0.72 t h^{-1}; Pressure=1 atm; T=25 ℃	Biomass used as combustion fuel
AIR1	Value=210 kmol h^{-1}; Pressure=1 atm; T=25 ℃	Air used in biomass combustion
AIR3	Value=50 kmol h^{-1}; Pressure=1 atm; T=25 ℃	Air used to cool biochar down
HOT-AIR4	Value=50 kmol h^{-1}; Pressure=0.81 atm; T=104.2 ℃	Hot air after heat exchange with biochar
AIR5	Value=160 kmol h^{-1}; Pressure=1 atm; T=25 ℃	Air used in pyrolysis gas combustion
EXHAUST2	Value=166.2 kmol h^{-1}; Pressure=0.15 bar; T=100 ℃	Exhaust stream after heat exchange with air
WATER1	Value=600 kg h^{-1}; Pressure=1 atm; T=25 ℃	Water used to cool down
HOTERWAT	Value=600 kg h^{-1}; Pressure=1 atm; T=81.3 ℃	Water output after heat exchange with pyrolysis vapor
C-FUME	Value=7662.1 m^3 h^{-1}; Pressure=1.0 bar; T=135.4 ℃	Exhaust fume after heat exchange with pyrolysis reactor
RECYFUME	Value=8580.8 m^3 h^{-1}; Pressure=1.01 bar; T=184.4 ℃	Heated vapor water after heat exchange with pyrolysis vapor
EXHAUST	Value=8765.7 m^3 h^{-1}; Pressure=1.0 bar; T=110.4 ℃	Exhaust stream after through the bag filter (B1)

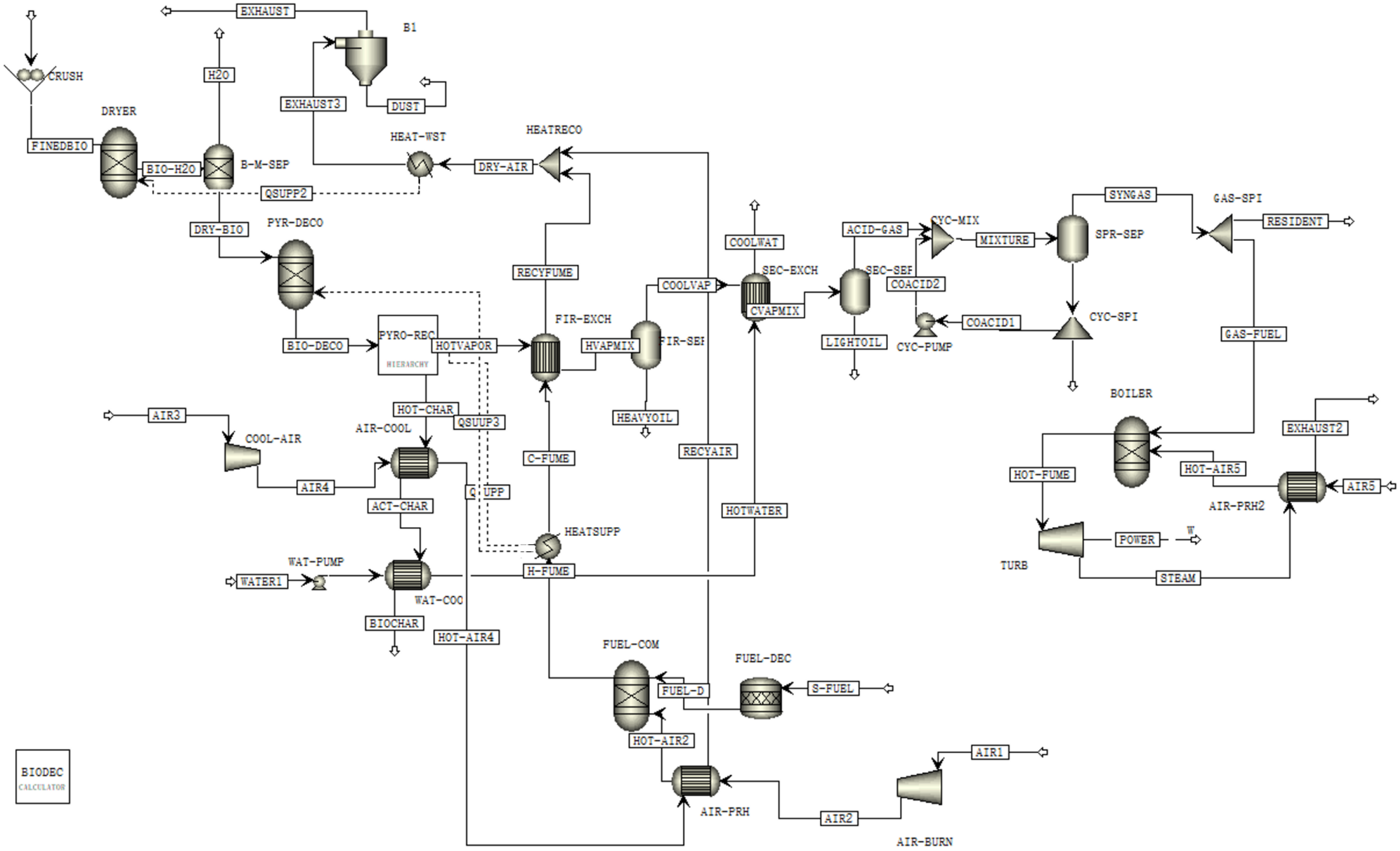

Supplementary Figure 1: Flowsheet for the biomass intermediate pyrolysis poly-generation process. The solid lines indicate the material flows, and the dashed lines represent the energy flows in the Aspen model.

Supplementary Note 3: Model inputs for Aspen Plus simulation

Pyrolysis kinetic equations

Debiagi et al.[1] proposed a detailed kinetic model for pyrolysis of biomass components to predict pyrolysis products. In this model, $(C_6H_{10}O_5)_n$ (xylan monomer, CELL) and $(C_5H_8O_4)_n$ (xylose-like cellulose monomer, HCELL) are set as the model compounds for cellulose and hemicellulose, respectively[3]. The chemical structure of lignin is relatively complex. It is composed in fact by LIG-C, LIG-H and LIG-O that are rich in C, H and O elements[4]. In the Aspen Plus simulation, the power law kinetic expression is used in the RCSTR reactor module. The formula is shown in Supplementary Equation (1):

$$r = k \times T^n \times exp(-E/RT) \prod (C_i)^{\alpha_i} \tag{1}$$

where *r* is the reaction rate, *k* is the pre-exponential factor, *T* is the absolute temperature, *n* is the temperature index, *E* is the activation energy of the reaction, *R* is the gas constant, C_i and α_i are the concentration and the partial order of reaction for i^{th} reaction educts, respectively.

Biomass component parameters

In this paper, rapeseed residue (crop residue) was selected as the raw material for testing the model. Results were compared with the experimental data in the literature[5] to verify the model validity. In addition, based on the composition parameters of eight major crop residue types (accounting for more than 90% of crop residues[6]) in China, a model compound was built and used for calculations in the Aspen Plus simulator. The composition parameters are shown in Supplementary Table 2.

Pyrolytic temperature and component conversion rate

In the pyrolysis reaction, the temperature of the flue gas used to heat the furnace gradually decreases from the bottom to the upper furnace. In this paper, the flue gas temperature is set to range from 250 °C to 650 °C. To simplify the heat exchange process, the biomass heating process is divided into 5 stages (PHASE1-5), and the temperature in each stage is increased by 100 °C.

Biomass components behave differently in each pyrolysis temperature range. Yang et al.[7] studied the pyrolysis process of biomass three components and suggested that the pyrolysis temperature range for cellulose is 315-400 °C, for hemicellulose is 22-315 °C, and for lignin is 400-600 °C. In order to simplify the reaction process, the reaction of hemicellulose (HCELL) occurs in PHASE1 and PHASE2, cellulose (CELL) reacts in PHASE2 and PHASE3, and lignin[8] reacts in PHASE3, PHASE4 and PHASE5. Based on the thermo-gravimetric experimental data[9] of rapeseed

residue, the conversion rates of the three components in each temperature range were set.

The secondary reactions settings for biomass pyrolysis

Biomass pyrolysis secondary reactions need a limit to their equilibrium conditions, in order to correct errors in the calculation (see Supplementary Table 5). In this study, the revised conditions are that the temperature of the RGibbs reactor is reduced by 200 °C.

Supplementary Table 2: Pyrolysis chemical reactions [1]

Reaction	k(s^{-1})	E(kcal mol^{-1})
Cellulose:		
CELL→CELLA	4×10^{13}	45
CELL→5H_2O+6Char	6.5×10^{7}	31
CELLA→0.45 HAA+ 0.2 Glyoxal+ 0.1 CH_3CHO + 0.25 HMFU+ 0.3 C_3H_6O + 0.15 CH_3OH + 0.4 CH_2O+ 0.31 CO+ 0.41 CO_2 + 0.1 H_2 + 0.83 H_2O+ 0.02 HCOOH + 0.2 CH_4 + 0.61 Char	2×10^{6}	19.1
CELLA→LVG	4T	10
Hemicellulose:		
HCE→0.5 HCE1+ 0.5 HCE2	1×10^{10}	31
HCE1→0.025 H_2O+ 0.775 CO_2 + 0.025 HCOOH+ 0.9 CO+ 0.8 CH_2O+ 0.125 C_2H_5OH + 0.55 CH_3OH + 0.25 C_2H_4 + 0.525 H_2+ 0.325 CH_4+ 0.875 Char	1.2×10^{9}	30
HCE1→0.25 H_2O + 0.95 CO_2+ 0.05 HCOOH+ 1.45 CO+ 1.4 H_2+ 0.3 CH_2O + 0.625 CH_4 + 0.375 C_2H_4 + 0.875 Char	0.15T	8
HCE1→XYLAN	3T	11
HCE2→0.2 H_2O+ 1.1 CO+ 0.675 CO_2 + 0.5 CH_2O+ 0.1 C_2H_5OH+ 0.2 HAA+ 0.025 HCOOH+ 0.25 CH_4 + 0.3 CH_3OH + 0.275 C_2H_4+ 0.925 H_2 + Char	5×10^{9}	33
Lignin:		
LIG→FE2MACR	4T	12
LIG→0.95 H_2O+ 0.2 CH_2O+ 0.4 CH_3OH+ 1.95 CO + 0.6 CH_4 + 0.05 HCOOH+ 0.5 H_2+ 0.65 C_2H_4 + 0.2 CH_3CHO + 0.2 C_3H_6O+ 5.5 Char	4×10^{8}	30
LIG→0.6H_2O+2.6CO+0.6CH_4+0.4CH_2O+0.5C_2H_4+0.4CH_3OH+2H_2+6Char	0.083T	8
LIG-C→0.35 LIG_{CC}+ 0.1 pCoumaryl+ 0.08 Phenol + 0.41 C_2H_4 + H_2O + 0.7 H_2 + 0.3 CH_2O+ 1.02 CO+ 0.495 CH_4+ 5.735 Char	1.33×10^{15}	48.5
LIG-H→LIG_{OH}+ 0.5 C_3H_6O+ 0.5 C_2H_4 + 0.25 HAA	6.7×10^{12}	37.5

LIG-O→LIG_{OH}+ CO_2	3.3×10^8	25.5
LIG_{CC}→0.3 pCoumaryl + 0.2 Phenol + 0.35 HAA+ 0.7 H_2O + 0.65 CH_4+ 0.6 C_2H_4+ H_2+ 1.8 CO + 6.75 Char	1.67×10^6	31.5
LIG_{OH}→LIG+ 0.9 H_2O+ 0.45 CH_4+ 0.9 CH_3OH + 0.95 H_2+ 0.05 CO_2+ 2 CO+ 0.05 HCOOH + 0.2 C_2H_4 + 4.15 Char	1×10^8	30
LIG_{OH}→1.5H_2O+6CO+1.75CH_4+4.4H_2+0.3C_2H_4+0.5CH_3OH+10.15Char	33	15
Extractives:		
TGL→ACROL+ 3 FFA	7×10^{12}	45.7
TANN → Phenol+ CO+ H_2O+ ITANN	20	10
ITANN→5 Char + 3 CO+ H_2O + H_2	1×10^3	25

Notes: In this Table, the abbreviations are an intermediate active cellulose (CELLA), hydroxyacetaldehyde (HAA, $C_2H_4O_2$), $C_2H_2O_2$ (glyoxal), 5-hydroxymethyl-furfural (HMFU, $C_6H_6O_3$), levoglucosan (LVG, $C_6H_{10}O_5$), hemicellulose (HCE), xylose monomer (XYL, $C_5H_8O_4$) sinapaldehyde (FE2MACR, $C_{11}H_{12}O_4$), paracoumaryl alcohol (pCoumaryl, $C_9H_{10}O_2$), C_6H_6O (phenol), triglycerides (TGL, $C_{57}H_{100}O_7$), free fatty acids (FFA), tannin (TANN, $C_{15}H_{12}O_7$), intermediate tannin (ITANN, $C_9H_6O_6$).

Supplementary Table 3: Biomass composition parameters as required by the reaction model[1,10,11]

Item	Composition	Rapeseed residues	Mixed crop residues [a]
Proximate analysis	Moisture	9.64	7.67
(wt. %), air dry basis	Ash	11.04	10.37
	Fixed Carbon	11.61	14.85
	Volatile matter	67.71	67.11
Ultimate analysis	C	46.12	43.83
(wt. %), dry basis	H	6.01	5.30
	N	1.76	0.61
	S	0.21	0.08
	Cl	0.00	0.35
	O	41.02	40.83
	Ash	4.88	9.00
Biochemical composition	CELL	42.61	44.24
(wt. %)	HCELL	34.59	30.02
	LIGH	1.80	0.51

	LIGO	10.72	12.73
	LIGC	0.68	1.41
	TGL	8.78	1.96
	TANN	0.82	9.13
Lower heating value (MJ kg^{-1})		14.14	14.70

[a] The mixed crop residues in this study refers to residues of eight main crops in China: rice, corn, wheat, soybeans, cotton, peanuts, rapeseed and sesame.

Supplementary Note 4: Model validation for Aspen Plus simulation

The simulation test investigated the pyrolysis performance of rapeseed residue in the condition of 250-650 °C and 1 atm, which was compared with the results of the three-state product pyrolysis of rapeseed residue in a packed bed[5]. As it is shown in Supplementary Table 6 and Supplementary Fig. 2, with comparison, the three-phase product distribution of the pyrolysis of rapeseed residue shows good agreement (error not exceed 6%) with the experimental results at the different operational temperatures. Especially in the reaction conditions of the medium-high temperature zone, the agreement is significant.

The major pyrolysis gas products of rapeseed residue are CO and CO_2. Due to secondary reactions, the contents of some gas products gradually increase with the increase of temperature, leading to an increase of the calorific value of the pyrolysis gas. Supplementary Table 4-6 shows the distribution of gaseous products of rapeseed residue pyrolysis at 650 °C. It can be seen that the experimental and simulation results display good agreement at this temperature. This is due to the fact that with the relatively high temperature, the pyrolysis secondary reactions are thoroughly complete and the residence time has little influence on the reaction results. Therefore, it is better to simulate this reaction with the restricted equilibrium RGibbs reactor module.

In summary, based on the biomass composition analysis and thermo-gravimetric analysis data, the pyrolysis model provided in this paper can be used to simulate biomass pyrolysis poly-generation.

Supplementary Table 4: The weight of three components for rapeseed residue in the pyrolysis process

Temperature (°C)	Biomass content (%)	The content of three components	
250	95	83% HCELL	/
350	45	1% HCELL	33% CELL

450	35	1% CELL	70% LIG
550	30	/	50% LIG
650	30	/	1% LIG

Supplementary Table 5: Secondary reactions settings

Phase	Component
Vapor	$C_2H_4O_2$ / CO / H_2 / CO_2 / H_2O / HCOOH / C_2H_4 / CH_4
Pure Solid	Char

Supplementary Table 6: The gas component distribution of rapeseed pyrolysis at 650 °C [5]

Component	Experiment (kmol t^{-1} biomass)	Errors (kmol t^{-1} biomass)	Simulation (kmol t^{-1} biomass)
CO	3.5	0.29	3.2
CO_2	2.1	0.23	2.6
H_2	2.7	0.29	2.1
C_2H_4	0.4	0.34	0.7
CH_4	2.2	0.26	1.8

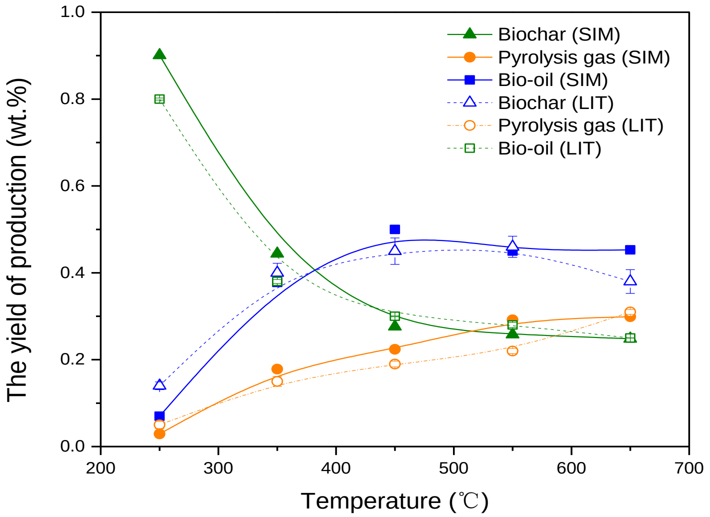

Supplementary Figure 2: The comparison of Aspen Plus simulation results and experimental data as a function of temperature. The experimental data are presented as mean values +/- Standard Deviation, n=3 independent experiments[5].

Supplementary Note 5: Analysis of biomass intermediate pyrolysis poly-generation (BIPP) material flows

The heat source of the BIPP system is derived from the high-temperature flue gases generated by the fuel combustion (i.e. biomass). In the simulation test, the heat required for decomposition of the biomass components and the components pyrolysis was calculated by the RCSTR dynamic reactor module. Results show that as the reaction temperature increases, the amount of heat required for the pyrolysis reactor increases correspondingly, as shown in Fig. 1.

The high-temperature biochar produced from the pyrolysis process needs to be cooled down rapidly to preserve its quality. The quality of biochar could be measured by parameters such as bulk density, ash content, surface area, volatile compound content[12]. This paper evaluates the quality from lower heating value (LHV) which has a close relationship with fixed carbon and volatile of biochar as shown in Supplementary Equation (2) and Supplementary Table 7 below.

In this paper, air-cooling and then water-cooling heat exchangers are used to decrease the temperature of the biochar to 100 °C, and the inputs required for air/water cooling are considered in the power consumption. Supplementary Fig. 3 shows the change of the cooling water consumption with the pyrolysis temperature. It can be seen that the trend of cooling water consumption is not monotonic along the temperature range and the trend is mainly influenced by the yield of biochar and by the difference between cooling temperature (100 °C) and pyrolysis temperature, both of which are shown in Supplementary Fig. 3. The trend of cooling water could be divided into three sections referred to by three temperature ranges:

a. 250 °C - 350 °C, the cooling water decreases dramatically due to the sharp decrease of the biochar yield with a temperature increase;
b. 350 °C - 450 °C, the cooling water consumption may be stable or decrease slowly and then increase, because of a similar change rate of biochar yield and temperature difference;
c. 450 °C - 650 °C, the cooling water increases gradually although the biochar yield has decreased at a relatively stable rate, which is lower than the rate of temperature increase.

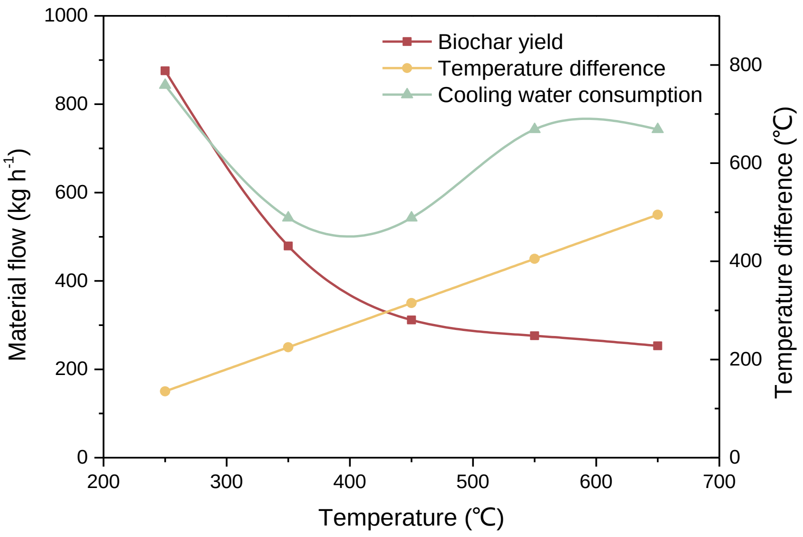

Supplementary Figure 3: Material flows, temperature difference between pyrolysis temperature and biochar output temperature. The material flows include biochar yield (red lines with square blocks) and cooling water consumption (green lines with triangle blocks), and the temperature difference is showed by yellow lines with circle blocks.

Supplementary Note 6: Analysis of BIPP energy flows

The energy inputs to the BIPP system are calculated based on the biomass feedstock input for pyrolysis and heat provision, as well as the electrical energy supplied to the system. Energy outputs are the sum of the energy content of the pyrolysis products (biochar, pyrolysis gas and bio-oil) and the energy loss during plant operation. The system energy efficiency is calculated for the energy transformation, which is defined as the useful energy recovered from pyrolysis products (i.e., pyrolysis gas, electricity, bio-oil and biochar) divided by the total energy input. The result of system energy efficiency has been compared with results for other pyrolysis systems in Supplementary Table 8.

The energy contained in the biomass input is calculated based on the LHV of the residue mixture shown in Supplementary Table 3. The energy produced from the pyrolysis products is estimated based on the LHV of biochar, pyrolysis gas and bio-oil.

The LHV of the biochar is sensitive to the content of ash, volatile matter, and fixed carbon in different pyrolysis final temperature. Based on experimental data[5] on the proximate analysis and the LHV of biochar, a fitted correlation can be obtained as shown in Supplementary Equation (2):

$$LHV = 31.74 - 0.14 \times Volatile - 0.11 \times C_{BC} \quad (2)$$

where *Volatile* is the volatile content (expressed in wt. %); and C_{BC} indicates the fixed carbon content of biochar (expressed in wt. %). According to the simulation results, the LHV of biochar can be calculated, as shown in Supplementary Table 7.

The LHV of pyrolysis gas is calculated based on its composition at various temperatures, with the results listed in Supplementary Table 7. Furthermore, given the complex composition of bio-oils, which could not be directly determined by the model, the LHV of bio-oils was considered to be 5.93 MJ kg^{-1}, based on the results of experimental tests and on those reported in the feasibility report[13] of an existing moving-bed BIPP plant.

Based on the above analysis and calculations, the energy flow diagram of BIPP under a constant temperature condition (550 °C) is shown in Supplementary Fig. 4. Energy transfer in the relative energy balance can satisfy Supplementary Equation (3):

$$Q_{in} = Q_{feedstock} + Q_{fuel} + Q_{power} = Q_{out} = Q_{products} + Q_{loss} = Q_{biochar} + Q_{pyro-gas} + Q_{elec} + Q_{bio-oil} + Q_{waste} \quad (3)$$

where $Q_{products}$ includes the $Q_{biochar}$, $Q_{pyro-gas}$, Q_{elec} (produced from the share of the pyrolysis gas that is fed to the gas turbine) and $Q_{bio-oil}$, all of which are based on the chemical energy content represented by the corresponding lower heating value; Q_{waste} indicates the waste heat from production and equipment of the BIPP system[14], which cannot be recovered in any way.

Supplementary Table 7: Energy performance of BIPP system (referred to 1 t of biomass feedstock input)

Parameter		Temperature (°C)				
		250	350	450	550	650
Biochar						
	Ash (wt. %)	13.57	27.52	44.25	47.33	49.21
	Fixed carbon (wt. %)	0.51	9.11	19.64	45.95	49.81
	Volatile (wt. %)	85.92	63.37	36.11	6.72	0.98
	LHV (MJ kg^{-1})	19.42	21.69	24.41	25.68	26.07
Pyrolysis gas						
	H_2 (kmol)	0.22	1.31	1.62	2.08	2.27
	CO (kmol)	0.30	1.89	2.65	3.57	4.00
	CO_2 (kmol)	0.21	1.42	1.72	2.51	2.54
	CH_4 (kmol)	0.08	0.59	0.75	1.89	2.00
	LHV（MJ $kmol^{-1}$）	230.54	234.06	240.95	284.16	287.00

Energy efficiency [a]	73.69%[b]	61.27%	50.89%	50.93%	48.75%

[a.] The system energy efficiency is calculated for the energy transformation, which is defined as the useful energy recovered from pyrolysis products divided by total energy input.

[b.] Given the errors of result in low temperature (explained in Supplementary Note 4), the energy efficiency at 250 °C is derived from the curve of efficiency drawn through other results ranging from 350 °C to 650 °C.

Supplementary Table 8: Energy performance of different technologies[14-16]

Study	Process type	Description	Energy efficiency
This study	Intermediate pyrolysis	A BIPP demonstration plant (250 - 650 °C)	48.75% - 73.69%
1	Slow pyrolysis	A continuous pyrolysis poly-generation system (550 - 650 °C)	82.1%
2	Slow pyrolysis	A pilot plant (550 °C)	58.9%
3	Fast pyrolysis	The pyrolysis system, including hot vapor filtration, a fractional condenser, and an electrostatic precipitator (500 °C)	61.01%

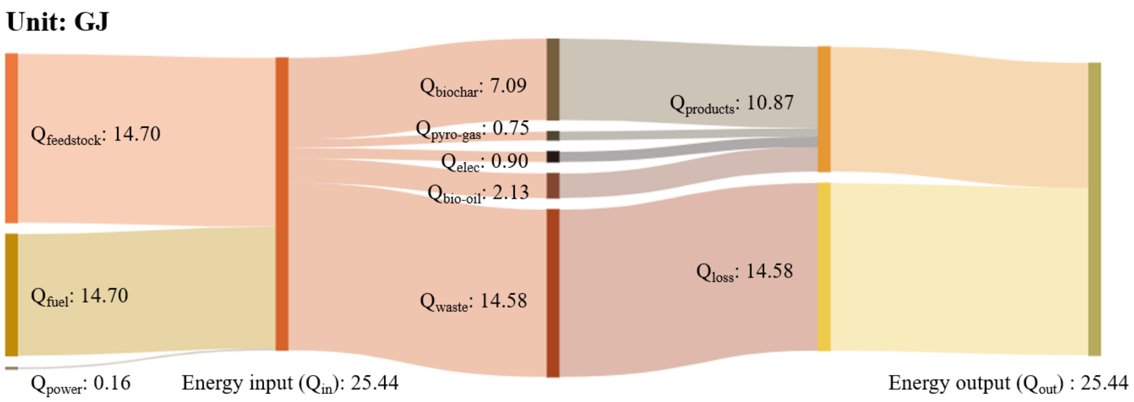

Supplementary Figure 4: Energy flow diagram of a BIPP system. The energy flows are based on the chemical energy content represented by the corresponding lower heating value under temperature of 550 °C for 1 t biomass feedstock input. $Q_{products}$ includes the $Q_{biochar}$, $Q_{pyro\text{-}gas}$, Q_{elec} (produced from the share of the pyrolysis gas that is fed to the gas turbine).

Supplementary Note 7: Economic benefit from pyrolysis production

All the cost values are calculated based on constant 2018 prices in USD. We converted RMB into USD with an average exchange rate (6.61 RMB USD^{-1} in 2018).

The biomass pyrolysis temperature can affect the quantity and quality of pyrolysis products, and further influence the economic benefits of the pyrolysis plant. It is important to correctly

understand the relationship between product yield, product quality (mainly referring to the heating value of pyrolysis gas in this paper) and the pyrolysis temperature when the economic benefits of this system is evaluated. The LHV of pyrolysis products have been discussed in Supplementary Note 7 and Supplementary Table 7.

Referring to 1 t of biomass feedstock input, the fitted relationship between pyrolysis inputs, outputs and pyrolysis temperature for BIPP system has been studied by the Aspen modeling. Thus, the effect of pyrolysis temperature on the overall economics of the system can be further investigated. Constrained by an undeveloped market for biochar and the lack of relevant policies for biochar production and sequestration in the soil, it is assumed that the biochar, used to improve soils for cultivation, was transported from the BIPP systems to the field directly. There is no profit from biochar selling from pyrolysis as assumed in previous research[17]. Thus the income are from the sale of pyrolysis gas, electricity and bio-oils (heavy and light oils) and carbon market. Limited by the current instability of the carbon market and price volatility of carbon price in China, the carbon price in EU-EST has been chose so as to explore the development of BIPP systems in future mature carbon market. It is assumed that the carbon price is 4.54×10^{2} USD t^{-1} (35€-40€ during 2020-2023 estimated by Carbon Tracker). The prices for these products are collected from the Chinese trading market reported on the website of Alibaba, the feasibility report of the Ezhou BIPP plant[11] and a previous biomass feasibility report[18]; in the feasibility reports the prices are set by the relevant BIPP company according to purchase and sale agreements with other companies. The prices are listed below (Supplementary Table 9).

Note that there has been a "Biomass Pyrolysis Cogeneration Joint Demonstration Project" operated by Ezhou Lanyan Biomass Energy Co., Ltd.in Hubei Province, operating since 2013, employing two sets of continuously moving bed reactors with a total capacity of 42700 t of crop residues per year[19], from which the market price of pyrolysis gas and bio-oil can be referenced. The benefit of a pyrolysis plant (B) can therefore be calculated as follows:

$$B = \text{Capacity} * \text{Hours} * (E_{red-BIPP} * 0.3 + Pyrolysis\ gas * P_{syn} + H_{oil} * 1.8 + L_{oil} * 2.0 + Power * 0.81) \quad (4)$$

where *Capacity* indicates the amount of biomass feedstock input consumed by the pyrolysis process, varying from 1 to 12 t per hour; *Hours* indicates the operational time for the pyrolysis plant, in this study assumed to be 5000 h per year; $E_{red-BIPP}$ is the GHG emissions reduction for a BIPP system; P_{pyrgas} is the price of pyrolysis gas; and the H_{oil}, L_{oil}, $Power$ are the quantity of heavy oil, light oil and electricity respectively.

Supplementary Table 9: The price of every production in pyrolysis plant[13,18,19]

Items	Price	Unit
Carbon price	4.54×10^{2}	USD t^{-1}
Biochar [a]	3.44×10^{2}	USD t^{-1}
Pyrolysis gas	4.54×10^{-2} / 6.81×10^{-2}	USD Nm^{-3}
Heavy oil	2.72×10^{2}	USD t^{-1}
Light oil	3.03×10^{2}	USD t^{-1}
Electricity	123×10^{-2}	USD kWh^{-1}

a. The price of biochar is used in sensitivity analysis.

Supplementary Note 8: Costs of the BIPP plant

To have an integrated economic-environmental assessment, an inventory of the costs of the whole process is required, including construction and operation and maintenance (O&M) phases, and the calculated equations have been shown in Methods.

On the consideration of the low energy densities and dispersed distribution of biomass feedstock, it is assumed that the BIPP plants are always built in locations where available biomass is abundant and easily supplied, just as was the case for the demonstration project in Ezhou, Hubei province. In this current demonstration plant, the biomass supply chain is organized by the local administrative authorities and the owner of the plant. Several collection stations are established around agricultural areas, where the dispersed biomass resource can be collected and stored, facilitating its purchase and transport to a BIPP plant. This study assumes that the biomass collection radius is 30 km as is described in the feasibility report. The collection cost is estimated in Supplementary Table 10 based on data derived from the literatures[20,21].

Supplementary Table 11 shows the parameters of the economic evaluation and financial indicators of these two components. In this table, Inside Battery Limit (ISBL) Costs refer to the purchase and installation costs of the major pyrolysis equipment components in the plant, while Outside Battery Limit (OSBL) Costs denote the costs of external transportation networks and storage facilities. The sources of initial investment costs in the table include feasibility study[13], while operating costs are mainly derived from the simulation results of the economic analysis module in Aspen Plus.

Supplementary Table 10: The biomass collection costs[20,21]

Item (USD t^{-1})	Purchase Cost	Transportation Cost	Other Cost [a]

Mixed crop residues	46	9	6

a. This includes the costs of biomass processing and storage.

Supplementary Table 11: List of economic parameters applied for the economic analysis [2,22,23]

Category	Parameters	Values	Explanation
Financing capital	Own Capital	0.3	
	Loan	0.7	
Investment	Interest Rate	0.0755	
	Depreciation	0.12	10 years to depreciate
	Discount Rate	0.08	
	Salvage Value	0.2	of the total capital investment
	Income Tax Rate	0.25	
	Additional Tax Rate	0.1	
	Capacity Factor	0.57	for 1 year
	Inside Battery Limits (ISBL) Costs		equipment costs + total installed costs
	Outside Battery Limits (OSBL) Costs	0.3	of ISBL costs
	Total Direct Cost		ISBL+OSBL costs
	Total Indirect Cost	0.2	of equipment costs
	Contingency	0.2	of direct and indirect costs
	Fixed Capital Investment		Direct + indirect + contingency
	Working Capital	0.05	of fixed capital investment
	Total Capital Investment		fixed capital + working capital
Operation costs	Raw Materials (RM)		Depends on capacity
	Operating Labor Cost [22]		simulation results
	Maintenance Cost [24]		simulation results
	Utilities (U)		simulation results
	Operating Charges (OC)	0.25	of total operating labor cost
	Plant Overhead (PO)	0.5	of total OLC+MC
	Subtotal Operating Costs		RM + OLC + MC +U+OC+PO
	G and A Costs	0.08	of subtotal operating costs
	Total costs		subtotal operating costs + G and A Costs

Supplementary Note 9: Economic indicator

As discussed above, the benefits and costs of a BIPP plant are affected by the inputs and outputs. The biomass raw material capacity and system operating temperature are the two most important factors affecting the economic benefits of the project. Based on the two-factor sensitivity analysis of net present value (NPV), the co-effects of the two factors on the economic benefits of a pyrolysis system have been inferred, and then the NPV has been calculated by Matlab R2018a.

NPV measures the profit resulting from the cash flows which happen during the entire lifetime of the project to the present moment. NPV can be determined by:

$$\text{NPV} = \sum_{t=0}^{n} (B_t - C_t - K_t)/(1+i)^t \quad (5)$$

where B_t and C_t are the benefit and cost in the tth year; K_t represents the investment in the tth year; and i indicates the discount rate.

In addition, the biomass price, one part of C_t calculation, varies with the change of areas. In the economic analysis the average prices for eight crop residues has been used. However, for the national economic analysis, the provincial average prices of eight crop residues have been collected from China's websites. Therefore, according to the Supplementary Equation (5), the NPV results per BIPP plant for 31 provinces have been calculated and shown in Fig. 4.

Supplementary Note 10: Life-cycle greenhouse gas emissions analysis for a demonstration BIPP system: Goal and scope

A primary goal of this study is to evaluate the net life-cycle greenhouse gas (GHG) emissions for the whole system.

The scope for hybrid LCA includes all material inputs required for the biomass cultivation and transportation, the construction of the demonstration BIPP plant (including equipment production), the O&M phases, the wastewater treatment neglected in previous studies[25], as well as the biochar utilization as soil amendment (including carbon fixation and biochar soil effect from biochar sequestration). The system boundary has been shown in Supplementary Fig. 5. Because of their minimal contribution to the final result of the study, GHG emissions associated with the electricity used in the plant construction and due to human labor are not taken into consideration. In addition, biochar used into soil has potential to improve the properties of the soil, such as an increase in soil water retention[26], pH, specific surface area[27], and a decrease of bulk density[28] and electrical conductivity[29], and is thus beneficial to crop cultivation and production. In this life-cycle

assessment, the biochar soil effect of enhancing crop production would not be considered in this life-cycle system boundary but would be taken into account in later scenario analyses. Thus, it is assumed that biochar could be used as soil amendment for farmers and in this scope just the GHG emissions from biochar soil effect could be considered.

The functional unit is 1 MJ energy produced for a demonstration BIPP system. The net life-cycle GHG emissions defines the life-cycle GHG emissions associated with transportation and the production processes of material inputs, subtracting the sum of carbon fixation from carbon sequestered in the form of biochar in soil and reduced GHG emissions from the biochar soil effect.

This study mainly considers the following greenhouse gases: CO_2, N_2O and CH_4. Referencing a time span of 100 years, IPPC reports equate 1 g of CH_4 and 1 g of N_2O with 25 g and 298 g of CO_2 respectively.

Supplementary Note 11: The life-cycle GHG emissions for the BIPP system

Indirect GHG emissions of crops in the cultivation phase

Indirect emissions define those produced during the development of inputs. In agricultural production, they result from the use of fertilizers, pesticides, machinery and diesel fuel. Given similar cultivation practices for the biomass (crop residue) types considered in this study, the pesticides, machinery and diesel fuel used in the cultivation processes are assumed to be the same for all biomass feedstocks. The fertilizers are classed as nitrogenous, phosphoric and potash fertilizers, as shown in Supplementary Table 13.

A market value method is used to allocate the GHG emissions between the main agricultural products and crop residue byproducts. In order to comprehensively assess the GHG emissions distributed between the main products and the byproducts of different crops, the allocation ratios has been estimated for the main crop types according to the Supplementary Equation (6):

$$Allocation\,ratio = \frac{P_{bypro} \times RG}{P_{bypro} \times RG + P_{pro}} \tag{6}$$

where the P_{bypro} and P_{pro} indicate the price of byproduct (i.e. the crop residues used as feedstock for BIPP) and product (i.e. grain) for one crop, respectively; and the *RG* is the relative ratio of crop residue to grain.

Direct GHG emissions of crops in the cultivation phase

For the direct GHG emissions during agricultural production, this study only considers the CO_2 emissions caused by the loss of soil organic carbon (SOC), the N_2O mainly associated with the input of nitrogen fertilizer into soil, and CH_4 emitted by anaerobic respiration of plant stems in paddy fields. Thus, CO_2, and N_2O emissions are considered in agricultural production for all crops, but CH_4 emissions are associated only with rice cultivation. The CO_2 emissions from loss of SOC (Supplementary Table 18) are significantly different for each province in China, and the change rates of SOC per unit of agricultural area[30] have been collected. According to the previous study[25], the N_2O emissions for different crops and CH_4 emissions from rice cultivation has been calculated, the total direct GHG emissions in cultivation phase have been shown in Supplementary Table 13.

This study first seeks to assess the life-cycle GHG emissions using a hybrid LCA method for one demonstration BIPP system. In order to simplify the model, it is assumed that the plant processes the residue of rapeseed, wheat and cotton as the main biomass feedstocks in Hubei province, based on an existing feasibility report[13]. Based on this the GHG emissions released during cultivation are reported in Supplementary Table 14.

Greenhouse gas emissions associated with transportation of biomass feedstock

Considering that the total benefits could be more than ten million, the capacity of the BIPP system is assumed to be 7.5t h^{-1} in the life-cycle assessment of the case study. The biomass materials processed by plant per year include 6.6×10^4 t agricultural residues (7.5 t h^{-1} biomass used in pyrolysis reaction and 5.7 t h^{-1} used as fuel to provide heat with 5000 operation hours). Fossil fuel is consumed and GHG emissions are produced during its transportation from collection stations to the BIPP plant. In this study, it is assumed that highway transport by diesel vehicles is adopted with an average transport distance of 40 km[25]. Thus, the GHG emissions associated with the transportation of biomass feedstock could be calculated according to the diesel consumption (the calculation method from the reference[25]).

Greenhouse gas emissions from the demonstration BIPP plant

The GHG emissions for the BIPP system include the emissions caused by the construction and O&M phases, as well as those caused by wastewater treatment. First, the costs of the construction and O&M phases have been calculated in the economic analysis. Then, by multiplying the costs for the emission intensity, the GHG emissions can be calculated according to the following Supplementary Equation (7):

$$E_{pyrolsis} = \sum E_i = \sum G_i \times MC_i \quad (7)$$

where E_i denotes the GHG emissions during the production of the ith input, and G_i is defined as the GHG intensity coefficient of the ith input (unit: t CO_2-eq 10^{-4} USD^{-1}), and MC_i indicates the money cost in the ith input with the unit of 1×10^4 USD. The estimated intensity coefficients were used for the year of 2017, by calculating and collecting from the carbon emissions and resources consumption reported in the Chinese economic statistics[31].

In addition, the CH_4 emissions associated with the wastewater treatment are an important source of GHG emissions caused by the operation of a pyrolysis plant. In the wastewater treatment system, anaerobic processes lead to CH_4, N_2O and CO_2 emissions, although N_2O is too negligible to be considered and CO_2 is not considered because of its biogenic origin[32]. According to the IPCC method[32], the CH_4 emissions for wastewater treatment has been calculated according to the following Supplementary Equation (8):

$$E_{CH_4} = \sum(TOW_i \cdot EF_i - R_i) \tag{8}$$

where E_{CH_4} represents the CH_4 emissions of the wastewater treatment; TOW_i represents the total organically degradable material in wastewater produced by industry *i* per year, expressed in kg of chemical oxygen demand (COD) per year; EF_i represents the emission factor for industry *i*, expressed in kg CH_4 $kg^{-1}COD^{-1}$; and R_i represents the amount of CH_4 recovered per year, expressed in kg CH_4 per year. At the time of the analysis year of 2018, China had not carried out large-scale recovery of CH_4, and as a result the default value of R_i is taken as zero. The CH_4 emission factor is therefore calculated as:

$$EF = \mathrm{B}_0 \cdot MCF \tag{9}$$

where B_0 represents the maximum CH_4 producing capacity, expressed in kg CH_4 $kg^{-1}COD^{-1}$, with a default value of 0.25 kg CH_4 $kg^{-1}COD^{-1}$; and MCF stands for the methane correction factor, with a default value of 0.5, based on IPCC and domestic research[32,33].

In this study, the wastewater is mainly comprised of cooling water and domestic water. Cooling water is first used to decrease the temperature of the biochar, is then used in the second-stage separator (water cooling tower), and finally goes into the gas purification and absorption system. The second-stage separator is a shell-and-tube indirect heat exchanger. A condensate collection channel is arranged at the bottom of the heat exchanger, so light materials and part of the condensate water of pyrolysis gas can enter the light oil tank. In the gas absorption system, water is used to wash the pyrolysis gas so as to remove the contained trace tar. The final water output is wastewater and processed in a wastewater treatment system. Limited by the Aspen block and in order to simplify the system, this study has simulated the gas purification and absorption system (CYC-MIX, CYC-SPI, CYC-PUM and SPR-SEP) without washing water flow and the wastewater

treatment system. Domestic water is daily water consumption by workers, which is ignored in the system diagram.

Nevertheless, the volume of the treated wastewater (cooling water and domestic water, shown in Supplementary Table 15) is calculated based on the feasibility report. Based on available data and Supplementary Equation (8) and (9), the CH_4 emissions associated with wastewater treatment are 37.0 t yr^{-1} (924 t CO_2-eq yr^{-1}).

Greenhouse gas emissions from the biochar used into soil

The role of biochar incorporated into soil includes two processes: the biochar transportation and introduction of biochar back into the soils of agricultural fields.

According to the simulation by Aspen Plus software, there would be 1.4×10^4 t biochar produced per year in the BIPP system. Given the immaturity of the biochar market and the lack of policies supporting biochar production and sequestration in soils in China, it is assumed that the biochar used in cultivation for improving soil is transported directly from BIPP systems to nearby fields (around 1600 km^2). Thus, the associated GHG emissions are emitted from the fossil fuel (i.e., diesel) consumption, and the calculation method is the same as the biomass feedstock transportation.

For the introduction of biochar back into the soils of agricultural fields, it is assumed that the biochar would be mixed with fertilizers and then used before farming. According to the literature[48], the application rate of biochar would be chosen as 20 Mg C ha^{-1} for the top 30 cm of soils which would have positive or neutral effects on biomass yield. However, given the immaturity of the biochar application method into soil, the biochar is seen as a mixed component of fertilizers so that the environmental impacts from the process of biochar introduction into the soils would be ignored.

Supplementary Table 12: Market allocation ratios for different crops in China[34].

	Ratio of crop residue to grain	Allocation ratio
Cotton	3	0.070
Wheat	1.1	0.142
Rapeseed	2	0.143
Rice	1	0.051
Corn	2	0.121
Soybeans	1.6	0.088
Peanuts	1.5	0.043
Sesame	3	0.032

Supplementary Table 13: The GHG emissions in the cultivation process for crop residues[35] (hm^2: hectometer squared)

Items	Fertilizers (kg hm^{-2})			GHG emissions (kg CO_2-eq hm^{-2})		
	Nitrogen	phosphorus	potassium	Indirect emissions	Direct emissions	Total emissions
Rice	163.5	64.5	43.5	22.2	217.5	239.7
Corn	165.0	60.0	31.5	49.9	16.1	66.0
Wheat	171.0	78.0	28.5	59.8	19.5	79.3
Soybeans	43.5	46.5	10.5	19.5	3.1	22.6
Cotton	183.0	76.5	61.5	34.9	10.3	45.2
Peanuts	73.5	61.5	70.5	16.4	2.5	18.9
Rapeseed	180.0	67.5	34.5	62.8	20.7	83.5
Sesame	99.0	58.5	39.0	11.2	2.5	13.7

Supplementary Table 14: GHG emissions in the life-cycle demonstration BIPP plant

Process	t CO_2-eq yr^{-1}
Agricultural production	1039
Transportation	593
Plant construction	3106
Plant operation and maintenance	1828
Water treatment	924
Total	7490

Supplementary Table 15: The CH_4 emissions from waste water treatment with 7.5 t biomass feedstock input per hour

Wastewater items	Quantity (kg h^{-1})	COD (kg L^{-1})[a]	CH_4 emissions (t yr^{-1})
Cooling water	4650	0.0125	36.5
Domestic water	2460[a]	0.0003	0.5
Total	7110	-	37.0

a. From feasibility report[11]

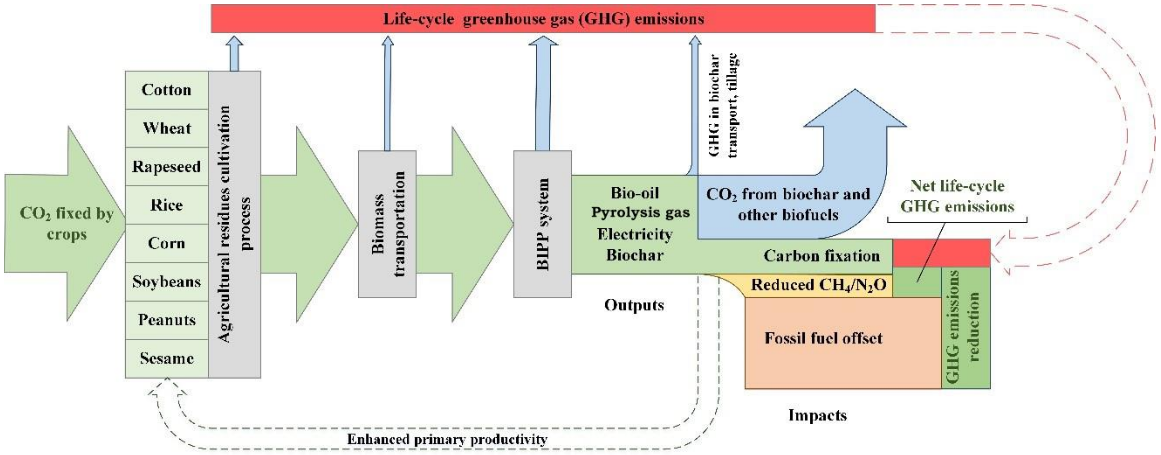

Supplementary Figure 5: Overview of the sustainable biochar concept and the system boundary of BIPP system with biochar sequestration. The green dotted line indicates the indirect relationship when the biochar is sequestered into soil and affects soil physical and chemical properties, enhancing primary productivity. The red dotted line shows the translation between the two red blocks, which are equal.

Supplementary Note 12: Carbon fixation in soil by biochar

It is clear that although the biochar yield declines with a temperature increase (Supplementary Fig. 2), the fixed carbon content in biochar goes up gradually. The sequestered carbon is directly associated with these two factors. In view of biochar characteristics following the temperature change as described below, the equation for carbon fixation in soil using biochar has been shown in Method.

The stability of biochar carbon is a critical factor affecting the net life-cycle GHG emissions of the system. In order to explore the differences of carbon fixation by biochar, therefore the experiments for various biomass feedstock in 600 ℃ by BIPP technology are made three times, and the O:C ratios of their biochar products are tested. The O:C ratio of biochar has been proposed as an indicator and defining threshold for the biochar stability in previous research[36,37]. This study referred to the research which used the accelerated ageing method to capture the physical correlation between stable biochar carbon and O:C ratio. The results have been shown in Supplementary Table 16. The results indicate that the biochar stability for eight crop residues ranges from 63% to 74%. Thus, for the demonstration BIPP system, according to the types and proportions of crop residues, it is assumed that 73% stable carbon would be considered as the base case in LCA analysis.

Supplementary Table 16: Biochar stability and carbon fixation for eight crop residues (NA: Not Available)

	O:C ratio	Biochar stability (%)	Carbon fixation of biochar (t per plant)
Cotton	0.05	73.3	1.46×10^4
Wheat	0.07	71.9	1.43×10^4
Rapeseed	0.07	73.3	1.46×10^4
Rice	0.08	71.2	1.42×10^4
Corn	0.20[a]	62.9	1.25×10^4
Soybeans	NA	66.8[b]	1.33×10^4
Peanuts	0.16	65.6	1.31×10^4
Sesame	NA	69.3[c]	1.38×10^4

a. The average value for the corn cobs and stalks;

b. According to reference [38]

c. The average value of biochar stability for seven agricultural residues.

Supplementary Note 13: The biochar soil effect of biochar sequestration

The biochar soil effect results from the changes in physical and chemical properties of the soil consequent to biochar additions. It is believed that biochar additions are beneficial in terms of reductions in soil N_2O and CH_4 emissions. Noting that there is controversy about the nature of the CH_4 emission reduction[39], thus, this study assumes that sequestration of biochar into soil has favorable impact only for N_2O emissions. There are also significant uncertainties associated with the reduced soil N_2O emissions for various biochar application rates (e.g. biochar quantity and applied depth) and soil condition (e.g. soil type)[40]. The annual avoided soil N_2O emissions (E_N) are calculated in terms of the following Supplementary Equation (10):

$$E_N = R_N \times (2.5 \text{ kg } N_2O \text{ ha}^{-1}\text{yr}^{-1}) \times A_b \tag{10}$$

where R_N denotes the reduction factor, which has been investigated in the range of 0 - 80% in previous research[40]. Given that this emissions factor has not been widely demonstrated especially for different conditions, this study assumes a modest value of $R_N =$ 60%; A_b indicates the area of land that has been amended by biochar. In this study the application rate of biochar is assumed to be equivalent to approximately 20 Mg ha^{-1} if incorporated to a depth of 0.3 m.

Supplementary Note 14: Reduced GHG emissions at a national scale

Assuming that this kind of BIPP system is applied throughout China, the GHG emissions reduction in every province can be assessed. Firstly, the spatial pattern of available crop residues is assessed as a preliminary guideline for the deployment of BIPP systems. According to the China Agricultural Yearbook (2014)[6], the total output of crop residues in China reached more than 800 million tons in 2014, of which more than 600 million tons can be collected and used, with a comprehensive residue utilization coefficient of 78%. In China, the National Development and Reform Commission has issued a crop residue Comprehensive Utilization Technology Catalogue (2014)[41], which claims that policy incentives are encouraging five uses of crop wastes: as fertilizer, as animal feed, as fuel, as a raw material to produce industrial materials (e.g. straw paper, purification material), and as a base material (i.e. cultivation base in the soilless cultivation production).

According to the current utilization of crop residues in China in 2014[42], biomass used as fuel accounts for about 11% of the total, as fertilizer about 35%, animal feed about 24%, industrial raw materials about 4% and base material about 4%. In addition to these five uses, around 22% of collected biomass is burned directly in the open air. In the base scenario, which assumes high levels of biomass utilization (which is not always compatible with utilization structure encouraged by the government in its biomass energy strategy), thus it is assumed that up to 33% of collected crop residues (11% used as fuel and 22% burned directly) can be used in China's pyrolysis projects in the near future. This means that it is necessary to try to discourage farmers from burning crop residues in the field so that they can instead be used to help achieve the national potential of GHG emissions reduction according to the Supplementary Equation (11):

$$E_{ij} = P_{ij} \times RG_i \times CR \times E_s \quad (11)$$

where E_{ij} indicates the amount of biomass feedstock in province *j* for crop *i*, including rice, wheat, rapeseed, cotton, peanuts, soybeans, corn and sesame; P_{ij} represent the annual yield; *CR* is the collection coefficient for residues; and E_s shows the percentage of biomass feedstock of the total available residues, in this study accounting for 33% as discussed above.

Based on this estimate of available biomass, the number of potential BIPP plants in every province can be calculated (Supplementary Table 17).

In addition, provincial environmental conditions will affect the GHG emissions associated with agricultural production. For instance, the fertilizer consumption for each kind of crop changes significantly (Supplementary Table 13). The direct GHG emissions of N_2O and CH_4 are influenced by the crop types, and direct CO_2 emissions are influenced by the soil types, precipitation and

ecosystem properties. Therefore this study assumes that the indirect and direct GHG emissions are affected by the crop types (Supplementary Table 13) and the distribution of soil types (i.e., SOC storage changes, see Supplementary Table 16) by province. The GHG emissions associated with agricultural production can be calculated considering the cultivated areas for different kinds of crops, according to China Agricultural Yearbook. The direct CO_2 emissions were calculated referring to the SOC storage changes in cropland of each province[30]. On the other hand, for the carbon fixation through biochar sequestration, this process would be influenced by biochar stability, which changes with crop types and pyrolysis conditions. In this study, the pyrolysis conditions are assumed to be those for BIPP systems with 600 ℃. The national distribution of GHG reduction potential could be assessed as shown in Fig. 4.

Supplementary Table 17: The available residues and corresponding potential BIPP plant in China.

City	Residue collection (kt)	Number of BIPP plants	City	Residue collection (kt)	Number of BIPP plants
Beijing	881.2	4	Henan	71728.3	361
Tianjin	2354.4	12	Hubei	29098.4	147
Hebei	41595.1	210	Hunan	28103.2	142
Shanxi	16234.9	82	Guangdong	11274.0	57
Inner Mongolia	38873.1	196	Guangxi	14333.9	72
Liaoning	26432.6	133	Hainan	1356.5	7
Jilin	49959.2	252	Chongqing	9679.6	49
Heilongjiang	78153.3	394	Sichuan	33508.6	169
Shanghai	887.4	4	Guizhou	10784.7	54
Jiangsu	32536.5	164	Yunnan	20254.3	102
Zhejiang	6263.7	32	Tibet	342.0	2
Anhui	36692.0	185	Shaanxi	14302.7	72
Fujian	4783.3	24	Gansu	12530.5	63
Jiangxi	18496.3	93	Qinghai	1122.2	6
Shandong	58387.2	294	Ningxia	4399.2	22
			Xinjiang	26164.8	132

Supplementary Table 18: Top ten provinces by loss of SOC per hectare, total loss of SOC, GHG emissions from agricultural residues, and GHG emissions reduction by BIPP deployments.

Ranking	Loss of SOC per hectare	Total loss of SOC	GHG emissions from agricultural residues	GHG emissions reduction
1	Inner Mongolia	Heilongjiang	Heilongjiang	Henan
2	Heilongjiang	Inner Mongolia	Hunan	Shandong
3	Jilin	Jilin	Henan	Heilongjiang
4	Liaoning	Henan	Anhui	Hebei
5	Xinjiang	Liaoning	Jiangxi	Anhui
6	Chongqing	Xinjiang	Jiangsu	Sichuan
7	Hainan	Shandong	Hubei	Jiangsu
8	Qinghai	Hebei	Sichuan	Hunan
9	Shanxi	Shanxi	Shandong	Hubei
10	Gansu	Shaanxi	Hebei	Inner Mongolia

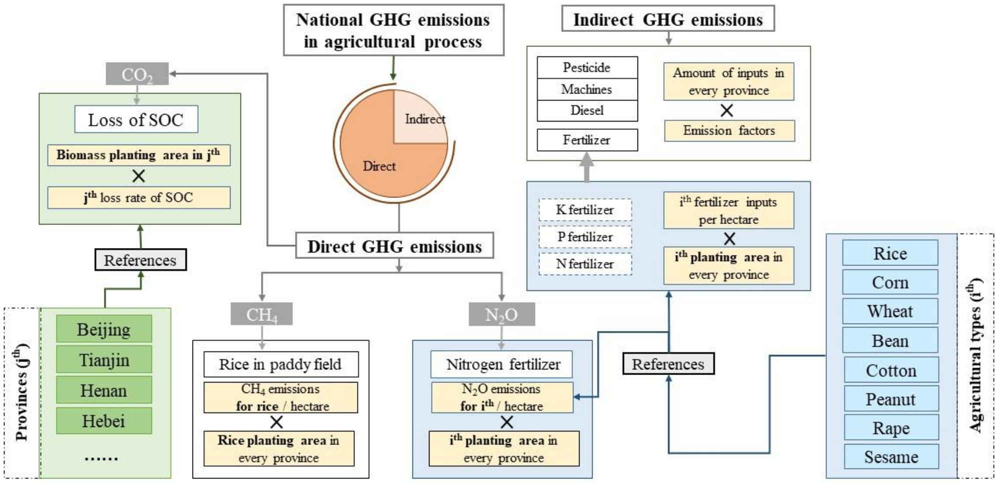

Supplementary Figure 6: Flow chart of national GHG emissions in agricultural process for BIPP plants. It includes the following greenhouse gases: CO_2, N_2O and CH_4. This calculation has considered the loss of soil organic carbon (SOC) for 31 provinces, except China Taiwan, Hong Kong and Macau due to the limited data.

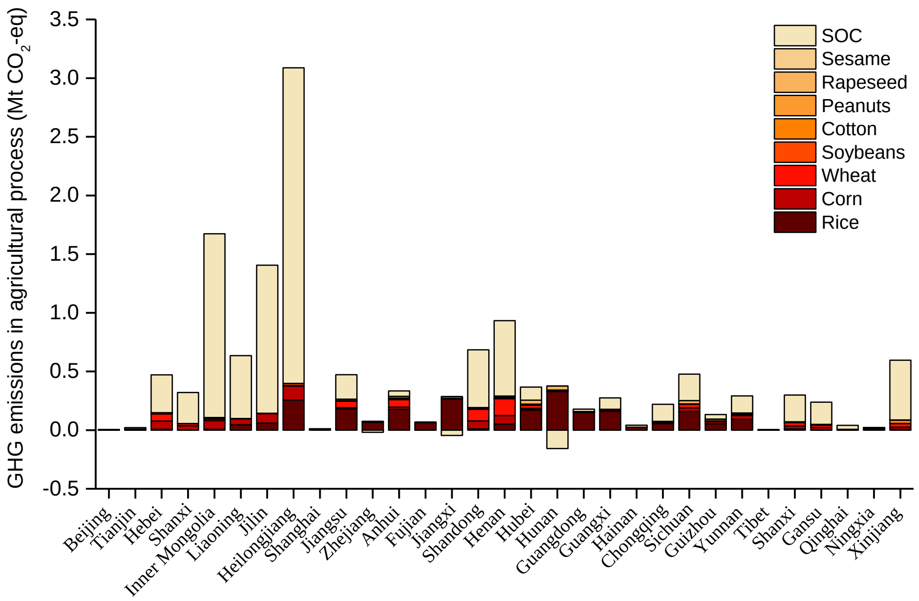

Supplementary Figure 7: GHG emissions of agricultural process by provinces. The GHG emissions have considered the CO_2 emissions associated with the distribution of crop species (i.e., rice, corn, wheat, soybeans, cotton, peanuts, rapeseed and sesame) and the CO_2 emissions caused by the loss of soil organic carbon (SOC) for different provinces.

Supplementary Note 15: Potential co-benefits for air pollution

Through the use of BIPP systems and the return and introduction of the biochar into agricultural soils, there are two beneficial impacts on the reduction of the emissions of air pollutants: one relates to the decrease of open-field and domestic biomass burning (OBB and DBB, respectively), and the second is associated with the emissions mitigated through the BIPP system.

Avoided air pollution from reduced OBB and DBB biomass burning

OBB and DBB is still a common way to dispose of crop residues after harvests in China. According to the above discussion, approximately 33% of available crop wastes are burned directly by households and in the fields, resulting in a large amount of air pollutant emissions (i.e., SO_2, NO_x, PM, VOC) released to the atmosphere[43,44]. The emissions from OBB and DBB can be estimated on the basis of factors as follows:

$$M_j = \sum_{i=1}^{8} P_i \times RG_i \times CR \times C \times EF_{ij} \quad (12)$$

where M_j refers to the air pollutants emitted from the direct burning of crop residues; P_i is the ith crop yield in every province (as illustrated above, with eight kinds of crops considered); *C* indicates the direct burning ratio for all crop residues with open and domestic burning ratios taken as 22% and 11%, respectively[42]; and EF_{ij} (g kg^{-1}) indicates the emission factor for corresponding air pollutants generated by burning the crop residues. Emission factors for OBB and DBB were derived from previous studies and are summarized in Supplementary Table 19.

Avoided air pollution from BIPP system

The avoided air pollution from BIPP system is the avoided air pollution from replacement of fossil fuels with BIPP products, subtracting the air pollution emissions from the combusted biomass stream used to generate heat for pyrolysis in BIPP system.

The BIPP system produces pyrolysis gas, bio-oil and biochar, the last sequestered into soil. For the pyrolysis gas, the LHV (shown in Supplementary Table 7) is higher than the minimum one required by CNS requirement (>7 MJ Nm^{-3}) for fuel gas for urban residents (GB 50028-2006). Considering the LHV and main components (i.e., CO, H_2 and CH_4), pyrolysis gas is similar with those of coke oven gas (COG, about 9 MJ Nm^{-3}), it is assumed that the pyrolysis gas could replace the COG for use by China's residents especially in the countryside. For bio-oil, it can be used as an alternative to coal tar after further purification and separation.

Because pyrolysis technology allows for removal of most particulates, mercury, and nitrogen and sulfur compounds, the utilization of pyrolysis gas in power generation and household heating/cooking can effectively reduce air pollutant emissions compared to supercritical coal-fired power plants (Sub-PC), combustion of COG and crude coal tar.

The air pollution emissions associated with the products of BIPP are allocated using a market-value method. The market prices are collected from a previous feasibility report[18], with the allocation shown in Supplementary Table 20. As a result, the emission reduction for air pollutant *k* by BIPP (M_{BIPP}^{k}) can be calculated as follows:

$$M_{BIPP}^{k} = \left(C_i^k - C_{BIPP}^{k}\right) \cdot E_{BIPP} - P_{bio-fuel} \times C_{bio-fuel}^{k} \quad (13)$$

where C_i^k, C_{BIPP}^{k} are the emission factors for air pollutant *k* associated with production of one unit of electricity, COG, coal tar, pyrolysis gas, and bio-oil (Supplementary Table 21); *i* represents respectively supercritical coal power plants, COG, or coal tar sources; E_{BIPP} represents the

products obtained by the BIPP (electricity, pyrolysis gas and bio-oil); and $C^{k}_{bio-fuel}$ indicates the emission factors for air pollutant k associated with combusted biomass fuel (unit: kg t^{-1} biomass fuel input) in BIPP system (data are from the feasibility report of the demonstration BIPP plant[13]); $P_{bio-fuel}$ represents the biomass combusted to generate heat for pyrolysis, k indicates the following air pollution species: SO_2, NO_x, primary $PM_{2.5}$ and BC.

Supplementary Table 19: Emission factors for biomass burning in open and domestic burning [45].

Sample	Domestic burning (kg t^{-1})				Opening burning (kg t^{-1})			
	SO_2	NO_x	$PM_{2.5}$	BC [a]	SO_2	NO_x	$PM_{2.5}$	BC [a]
Rice	0.53	0.42	1.66	0.066	0.53	1.29	9.65	0.37
Wheat	0.53	0.86	5.61	0.22	0.85	3.30	7.60	0.30
Corn	0.53	0.76	2.45	0.098	0.44	4.30	11.7	0.47
Cotton	0.53	1.29	6.04	0.24	0.53	1.29	9.65	0.39
Others	0.53	1.29	3.94	0.16	0.53	1.29	9.65	0.39

a. The emission factor of BC is calculated based on the emission factor of $PM_{2.5}$.

Supplementary Table 20: The air pollution allocations of BIPP products for per t biomass feedstock input in 600 ℃ (market-value allocation method).

Item	Output (kg h^{-1})	Cost (USD)	%	SO_2	NO_x	$PM_{2.5}$
Biochar	269.06	93	41.22	145.80	413.06	37.08
Pyrolysis gas	282.71	64	28.56	101.01	286.16	25.69
Bio-oil	329.01	65	28.80	101.88	288.62	25.91
Acid	13.992	3	1.41	5.00	14.16	1.27

Supplementary Table 21: GHG and pollutant emission factors from power plants[24,46,47].

	Supercritical coal-fired power plant (Sub-PC, g kWh^{-1})	Coke Oven Gas (COG, g Nm^{-3})[a]	Coal tar (g t^{-1})[a]	Charcoal (g MJ^{-1})[b]	BIPP systems			
					Electricity (g kWh^{-1})[c]	Pyrolysis gas (g Nm^{-3})[c]	Bio-oil (g t^{-1})[c]	Emissions from the combusted biomass (kg t^{-1})[d]
SO_2	0.331	0.300	1098.07	-	0.289	0.357	309.66	0.32
NO_x	0.968 (0.773-1.583)	0.106	388.70	-	0.819	1.012	877.24	1.02

$PM_{2.5}$	0.168 (0.116-0.236)	0.178	651.36	-	0.073	0.091	78.85	9.73
GHG (CO_2-eq)	747.55	443.19	1622806.20	107	-	-	-	-
LHV (Unit)	-	9.13 MJ Nm^{-3}	33.45 MJ kg^{-1}	-	-	13.69 MJ Nm^{-3}	5.93 MJ kg^{-1}	-

a. Adopting energy content allocation method for coal tar which is a byproduct of coke production.

b. The charcoal production is used in metallurgical and other industries, it can be substituted by biochar in alternative scenario (seen in Life-cycle GHG emissions for a demonstration BIPP system).

c. The avoided air pollution from replacement of fossil fuel with BIPP products.

d. According to the feasibility report[13].

Supplementary Table 22: Pollutant emission reductions per BIPP plant

	Electricity (g h^{-1})	Pyrolysis gas (g h^{-1})	Bio-oil (g h^{-1})	Total (kg yr^{-1})
SO_2	88.2	-109.0	1945.5	9624
NO_x	312.9	113.6	-1205.5	-3895
$PM_{2.5}$	199.5	49.2	1412.7	8307
BC	7.89	1.97	56.5	332

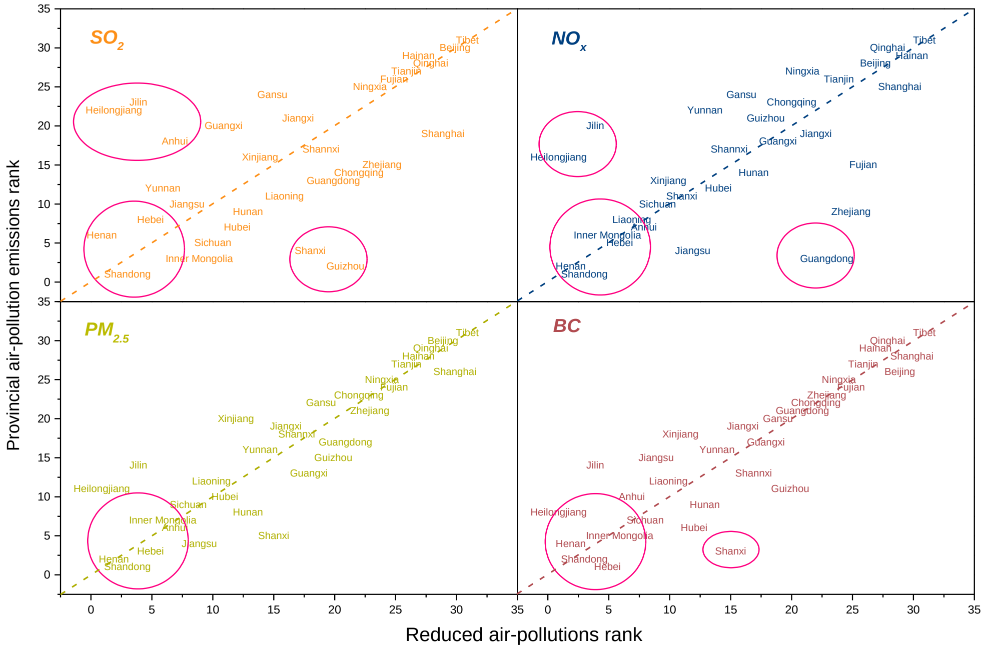

Supplementary Figure 8: The relative ranks for annual reduced air pollutions (SO_2, NO_x, $PM_{2.5}$, and BC) and provincial air pollution emissions. The diagonal indicates the 1:1 weight coefficient for these two aspects.

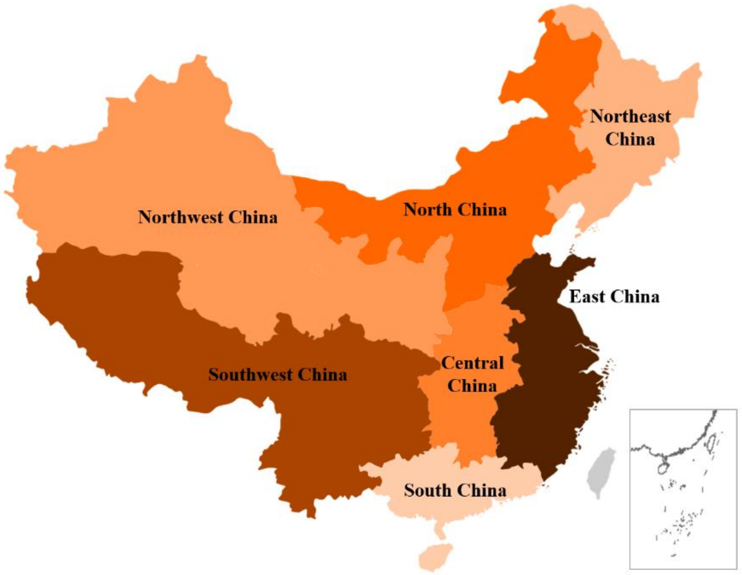

Supplementary Figure 9: The regional divisions in China. The China Taiwan, Hong Kong and Macau do not be considered in this map.

Supplementary Note 16: Influence of biomass availability for BIPP deployment

Deployment of negative emission technologies (NETs) and GHG emissions reduction were first considered in climate change mitigation scenarios exploring feasible strategies to limit the rise in average surface temperature to 2 °C in the IPCC's Fourth Assessment Report (AR4)[48]. According to a set of initial models, this report concluded that the NETs would have to play an essential role in future mitigation targets. Bioenergy with carbon capture and storage (BECCS) is the only NET included in the scenarios of IPCC and prompted widespread interest[49]. The special report from IPCC[50] emphasizes the potential of BECCS in the future, but points out trade-offs with other constraints such as total biomass production that should be considered for regions with spatially heterogeneous sources of biomass.

This study focuses on China's unique distribution, exploring the development of bio-energy NETs—biochar from BIPP and BECCS—based on the limits of available biomass.

Supplementary Note 17: Scenarios of bio-energy NETs deployment during 2020-2050

For now, although BECCS theoretically can sequester larger fractions of carbon from biomass into stable reservoirs compared with biochar[51], this technology currently faces many technological[52], economic[53] and environmental bariers[54]. Thus, based on the research status, as well as in accordance with scenarios analyzed in the IPCC reports, it is assumed that it can be deployed at large-scale starting in 2030. Conversely, the pyrolysis technology has been developed for decades and demonstration plants are already operational in China. An increasing development of pyrolysis technologies is thus likely in the near future. In these scenarios, it is assumed that biochar can be applied nationally from 2020 to mitigate the potential overshooting of the GHG budget in the first half of this century[55,56].

Two groups are considered for scenarios for analysis of China's biomass availability and utilization:

- Group 1 – Scenarios labeled "Moderate development of bio-NETs" assume biochar and BECCS deployment in different sustainable biomass allocations that consider China's current practices and policies concerning the collection and utilization of crop residues;
- Group 2 – Scenarios labeled "Maximum bio-NETs potentials" assume extreme conditions aimed at maximizing China's contributions to climate change mitigation by exploiting the full potential of its biomass resources.

Dealing with the evaluation of biomass availability in the near future, studies of future food

production trends have recently developed a set of various models that propose scenarios under changing factors, such as future population, climate change, water use, and energy structure[57-60]. However existing studies have concluded that there is a high degree of uncertainty in future agricultural production, which is sensitive to climate change, land degradation, and other factors. Furthermore, published reports[61,62] from China claim that given that the main crop yields, cultivation practices and residues use in China have not changed fundamentally in recent years, it is expected that there will be no significant changes in the yield and possible utilization of agricultural resources in the next few years. Thus, this study makes a simplifying assumption that China's agricultural resources and residues utilization will remain at the same level as the present situation in the first half of the century, and the impacts of biomass availability under various scenarios with changing factors are beyond the scope of this study, except for biochar use as a soil amendment.

Crop residues in China are encouraged to be used efficiently as discussed in Supplementary Note 14. As a fodder livestock, biomass can expand the feed resource availability and save food, a significant consideration in biomass utilization[63]. Compared to the simple handling and then low energy transformation of China's biomass utilization, developed countries have higher efficiencies of biomass use, thus their crop residues used for feed is less than 20% of all biomass but can satisfy large demands for livestock operations. Accordingly, it is assumed that there is up to 11%, 33% (base case, the share currently used in energy systems and burned in fields), 73% (all except the biomass used in industry and for animal feed) and a maximum of 80% of available crop residues (all except crop residues used for animal feed assuming higher efficiency) that could be used for BIPP deployment with biochar sequestration.

Pressing climate-mitigation challenges require us to get underway soon efforts to launch in the market negative CO_2-emitting technologies, and BIPP technology is a good choose in near future. This means that there will be competition for biomass feedstock between biochar and BECCS if China plans to deploy massive BECCS systems at the beginning of 2030 to achieve this goal of annual reduction emissions. Based on this consideration, this study developed three BECCS-BIPP-substitution scenarios to estimate the 'moderate' development of bio-NETs. These scenarios assume that the maximum available biomass is only 73% for all available crop residues over the period 2020-2030, and that by 2050 this figure could increase to 80% as discussed above.

In addition, it is assumed that the calculations for the scenarios analysis during 2020-2050 are based on the comparison with the fossil fuel technologies in 2017 (has been considered in Supplementary Note 14), which is described as Business-as-Usual (BAU) in this study.

Supplementary Note 18: Introduction of BECCS in scenarios

BECCS can be used for various applications, including power generation, industrial use, and fuel production. However, due to the technological constraints and economic barriers, there are currently only five operating BECCS programs worldwide[49]. All the projects apply CCS combined with ethanol production, with relatively low investment costs and high efficiency. Given the limited market and unsustainability of bio-ethanol in China[64], however, it is projected that BECCS to produce ethanol will not be widely deployed in future. Thus, this paper assumes that biomass gasification power plants are coupled with CCS in the future[65], as suggested in the latest report[66] in meeting the growth of power demand and reducing carbon emissions. In addition, the BIPP systems can be transformed into biomass gasification power plants with CCS systems at various substitution rates through retrofitting of equipment (e.g., reactors) and changes of key reaction parameters (e.g., temperature, reaction atmosphere)[65] and adding CCS technology to flue gas outlets, lowering capital investments and time requirements to achieve large-scale BECCS deployment. Moreover, our previous study also showed synergies between BECCS and biochar, by using ash from wood combustion, a byproduct from BECCS, as an additive in biochar production, which can significantly increase the carbon sequestration potential of biochar and decrease the CO_2 abatement costs[67]. In a conservative estimation, the synergies are not considered in this study. The detailed information about the scenario assumptions have been explained in Method.

Accordingly, given data shortages for BECCS in China, various references were collected and the GHG intensities for every sub-system of bioenergy gasification power plants coupled with CCS were used to evaluate the potential for GHG emissions reduction. To facilitate comparison of bio-energy NET and narrow the data differences across their life cycles, this study adopted the same hybrid LCA method to calculate the GHG emission intensities in construction and operation of the gasification power plant as below (Supplementary Table 23). For both technologies, the analysis is based on the functional unit of produced energy (MJ), and assumes that they are based on the same biomass supply, which is in independent of NET technologies. The parameters for the BECCS plant are shown in Supplementary Table 24.

Supplementary Table 23: GHG emissions intensity for Sub-PC and biomass gasification with CCS plant (OC: own calculation) [24,68-71]

	GHG intensity (g CO_2-eq kWh^{-1})[a]	Without CCS	With CCS [b]
Sub-PC		747.6	-

Biomass gasification with CCS plant	Biomass transportation	7.2 [OC]	8 [OC]
	Construction phase	401.8 [OC]	446.4 [OC]
	O&M phase	15.6 [OC]	17.3 [OC]
	CO_2 capture	-	55
	CO_2 compression and transport	-	1.8
	CO_2 injection	-	7.1
	CO_2 stored		-1109

a. This includes all the sub-systems of the biomass gasification power plant with CCS except agricultural production.

b. Research works[72,73] demonstrate that there is a decrease in system's efficiency when combining CCS into bio-energy conversion technologies. Accordingly, it is assumed that efficiency drops down of 10%.

Supplementary Table 24: Important parameters of the biomass gasification plant with CCS in China [18]

Items	Values
Biomass demand	1.73×10^5 t yr^{-1}
Electricity production [a]	9.81×10^7 kWh yr^{-1}
Net life-cycle GHG emissions intensity	-573.4 g kWh^{-1}
GHG emissions reduction intensity	1321.0 g kWh^{-1}
Total net life-cycle GHG emissions (except agricultural production)[a]	-0.056 Mt
Total reduced GHG emissions (except agricultural production)[a]	0.130 Mt

a. Calculated with the assumed efficiency drops down of 10%.

Supplementary Note 19: Introduction of energy crops in the scenarios

Energy crop cultivation offers another option to narrow the gap between biomass feedstock demand and supply[74] in future China. Miscanthus (commonly known as silvergrass) is regarded as a leading candidate for second-generation energy crops due to its high biomass production potential and low requirements for inputs during growth. The available land is the primary constraint for bioenergy development. This study therefore evaluated the energy potential for Miscanthus in marginal land without threatening food security by Geographical Information System software (ArcGIS)[74]. In addition, the effect of biochar sequestration in marginal land is added (see Supplementary Table 25).

The results indicate that the most marginal area that can contribute to Miscanthus cultivation amounts to 226,000 km^2 with the maximum yield of 343 Mt per year (harvesting 15.2 t $ha^{-1}yr^{-1}$).

On the consideration of the little active practice in the use of marginal land to generate energy crops[75], in the scenarios of 'Maximum biochar potential', this study assumes that the Miscanthus cultivation increases with a growth rate of 7793 km^2 per year, from almost zero in 2020 to the largest conceivable yield in 2050.

Supplementary Table 25: GHG emissions for Miscanthus production[76]

Stage of production	GHG emissions (kg CO_2-eq) released form per t Miscanthus
N_2O emissions from fertilizer and crop residues	0.14
Fertilizer and pesticides	56.35
Field operations	12.83
Transport	27.78
Carbon sequestration (CO_2) [a]	-131.00
Total	-33.90

a. It is related to the above and below ground crop residues of Miscanthus.

Supplementary Note 20: Introduction of forest residues in the scenarios

The national forest area of China amounts to 208 million hectares and the forest coverage rate is 21.63%. Therefore, forest residues could provide a significant biomass resource or input for bio-energy utilization. Given that the forest sector in China is characterized by a great diversity of forest types[77], this study further evaluated the available forest residues in China classifying them into four types of biomass resource: forest management residues, forest harvesting residues, timber processing residues and bamboo processing residues. The results of available forest residues have been shown in Supplementary Table 26.

Supplementary Table 26: Available forest residues in China[78,79]

			Forest residues (Mt)
Timber processing residues	Processing wood (m^3)	4.61×10^7	17.10
Forest harvesting residues	Commercial wood ($m^{3)}$	7.78×10^7	38.92
Bamboo processing residues	Bamboo production (root)	2.52×10^9	23.47
Forest tending residues	Timber stands (hm^2)	6.72×10^7	12.61
	Firewood forests (hm^2)	1.77×10^6	66.38
	Protection forests (hm^2)	9.96×10^7	74.75

	Economic forests (hm²)	2.06×10^7	3.86
	Special-use forests (hm²)	1.63×10^7	3.06
Total			353.61

Supplementary Note 21: Discussions of modelling scenarios for China's bio-NETs deployment

This study made a conservative but detailed assessment for the potential of bio-NETs by mid-century. Thus, it is believed that the China's bio-NETs deployment can play a more important role in the temperature-limited target for the reasons outlined below:

a. As has been discussed, agricultural production in the future has a high degree of uncertainty influenced by future population, climate change etc., and China claimed that there are no significant changes in agricultural resources in near future. Therefore, this study assumed conservatively that China's agricultural resources and residues utilization would remain at the same level as the present situation in the first half of the century. However, the report published by the Food and Agriculture Organization (FAO) suggests that on consideration of the increased population and changes in diet, agricultural production will have to rise significantly to meet the demand by 2050[80]. Thus, the bio-NETs could have a larger potential of GHG emissions reduction and negative emissions given the prospect for agricultural production growth in future.

b. This study has analyzed the very detailed inventory inputs for a demonstration BIPP system, including construction processes (e.g. equipment and installation) and wastewater treatment (i.e. water use and CH_4 emission) which have been ignored in previous studies[31,32]. In addition, this study has also accounted comprehensively for the national environmental effects with high spatial resolution for the optimal distribution of agro-energy systems (e.g., crop types, yields) in different geographic regions (considering also soil status). However, some of the global reduction goals for BECCS to meet the 2 ℃ /1.5 ℃ target in the IPCC report were investigated and calculated by assuming carbon neutrality for biomass technologies[81]. Thus, it is believed that this study has made a more detailed assessment. Thus, China's bio-NETs can make a larger contribution in this century to the global goals for BECCS claimed in IPCC.

c. The static carbon intensities of various sectors (e.g., steel, electricity sector) were applied in calculations in order to simplify the assessment. However, contributed by countries

policies (e.g., renewable energy incentives) and technological developments, the carbon intensities of sectors would decrease gradually over time. And the decreased carbon intensities could lower the GHG emissions for upstream production and services over the whole life cycle, causing a decrease in GHG emissions for targeted systems (BIPP system in this study). Thus, the negative emissions could be larger if the dynamic carbon intensities were considered.

d. This study aims at the biochar application produced from agro-biomass (mainly agricultural residues, including also to some extent scenarios) in BIPP systems involving forest residues and energy crops. However, the wastes (e.g., manures, municipal solid waste) also involve significant sources of biomass with potentially large availability for pyrolysis systems[40,82], but were not considered in these modelled scenarios. Therefore, it is believed that the China's bio-NETs could play a more significant role in the global reduction goal when the various biomass feedstocks, including agro-biomass and wastes, are included.

Supplementary Note 22: Sensitivity analysis of the economic feasibility of a demonstration BIPP system

The different scenarios used in sensitivity analysis of economic feasibility of the different demonstration BIPP plant scenarios, due to the change of key factors, including pyrolysis gas distribution, bio-oil price, carbon price and biochar usage, are presented in Supplementary Table 27. Key assumptions for these scenarios are discussed below, and the results are shown in Supplementary Fig. 10.

The results indicate that the NPV of a demonstration BIPP system can vary widely under different scenarios (from -1.70×10^{7} to 1.66×10^{9} USD; the base case is 2.44×10^{6} USD) under the different scenarios. Especially under the 1.5/2 ℃ temperature control target (scenario C), the carbon price could reach as high as 6050 USD_{2010} t^{-1} [50] and then the NPV would achieve 1.66×10^{9} USD. This high profit potential would help the system dramatically enhance economic competitiveness in the energy market. The bio-oil price will also change the NPV significantly. For other scenarios regarding changes of pyrolysis gas use distribution and biochar usage, the NPV does not change dramatically. It is concluded that the economic feasibility is more sensitive to the prices of products, than to pyrolysis gas use distribution and biochar usage. Therefore, a stable and mature market is a prerequisite to ensure the stable development and application of the BIPP system.

Scenario A. Pyrolysis gas distribution

In the base case simulation, according to the feasibility report of the demonstration BIPP plant, 20% of the pyrolysis gas produced by the BIPP plant is provided for household heating and 80% is used for power generation. In the sensitivity analysis, two extreme cases were simulated: 0% pyrolysis gas for household heating + 100% pyrolysis gas for power generation, and 80% for household heating + 20% for power generation (shown in Supplementary Table 27). Note that reserving 20% of the pyrolysis gas for power generation limit is determined by the auxiliary power consumption of the demonstration BIPP plant.

Scenario B. Price of bio-oil

Bio-oil is an important co-product from the BIPP system. In the base case, the price of bio-oils (heavy and light oil) is the average value in the Purchase and Sale Agreement of the Ezhou BIPP Plant that this particular BIPP company sets with other companies. However, it is believed that the price of bio-oils could vary with the change of quality under different reaction condition, and also could vary over time in the market. Considering the complexity and difficulty of predicting bio-oils price, they are assumed to vary according to the fluctuation of crude oil price.

As of June 2020, the average annual price of crude oil for 2020 stood at 39.89 USD per barrel. Between 1998 and 2020, the lowest and highest crude oil price reached 12.8 (1998) and 111.63 (2012) USD per barrel, respectively[83]. The change range of crude oil price is thus calculated to be -67.9% - 179.8%. Accordingly, this range is applied to the bio-oil price in the sensitivity analysis (shown in Supplementary Table 27).

Scenario C. Carbon price

A carbon market can be an important tool in carbon reduction. In the base case, the carbon price in the European Union Emission Trading Scheme (EU-EST) is chosen to assess the deployment of BIPP systems in a mature carbon market. In order to explore the development potential of BIPP systems in the future, a range of carbon prices under the framework of the Paris Agreement has been considered (Supplementary Table 28) in the sensitivity analysis.

Scenario D. Biochar usage

The biochar sequestration in soil would result in negative carbon emissions, from which BIPP plants could benefit in the carbon trading market. 100% biochar is sequestered in soil in the base case. However, in feasibility report of the demonstration BIPP plant, biochar is also recommended

to be sold as solid fuel in industrial market. The biochar usage in industrial market could bring different economic benefits. In the sensitivity analysis, two cases are analyzed to consider the potential economic benefits (NPV): 60% biochar for soil sequestration + 40% biochar selling in the industrial market; and 100% biochar selling in the industrial market (shown in Supplementary Table 27).

Supplementary Table 27: Scenarios for sensitivity analysis

Scenarios		
Economic feasibility		
A (Pyrolysis gas distribution)	Lowest	0% household gas + 100% power generation
	Highest	80% household gas + 20% power generation
B (Price of bio-oil)	Lowest	Price after change rate of -67.9% for base case
	Highest	Price after change rate of 179.8% for base case
C (Price of carbon price)	Lowest	Carbon price -15 USD_{2010} t^{-1}
	Highest	Carbon price -6050 USD_{2010} t^{-1}
D (Biochar usage)	Highest	40% biochar selling + 60% biochar sequestration in soil
Life-cycle GHG emissions		
E (Biochar stability)	Lowest	Biochar stability -80.0%
	Highest	Biochar stability -60.0%
F (Transport distance)	Lowest	Average distance for biomass feedstock -20 km
	Highest	Average distance for biomass feedstock -100 km
G (Transport method)	Lowest	Biomass transport by electricity vehicles
	Highest	Biomass transport by gasoline vehicles
H (N_2O reduction from biochar soil effect)	Lowest	Reduction factor for N_2O emissions (R_N) -80%
	Highest	Reduction factor for N_2O emissions (R_N) -0%
I (Biochar usage)	Highest	40% biochar selling + 60% biochar sequestration in soil

Supplementary Table 28: The carbon price required to achieve the global temperature control target under the framework of the Paris Agreement [50,55]

Source	Price (USD_{2010} t^{-1})	Year	Target
IPCC, 2014	18-250	2020	2℃
IPCC, 2018	15-6050	2030	1.5℃

Supplementary Note 23: Sensitivity analysis of the life-cycle GHG emissions for a demonstration BIPP system

In order to explore the sensitivity of the life-cycle GHG emissions of the demonstration BIPP plant, scenarios with variations of the key factors, including biochar stability, transport models and distances, N_2O reduction and biochar usage, are analyzed and listed in Supplementary Table 27. Key assumptions for these scenarios are discussed below. The results are shown in Supplementary Fig. 10.

It is found that the most sensitive factors affecting the BIPP's life-cycle GHG emissions are closely related to biochar, including biochar stability (scenario E, -19.7% to 34.6% relative to the base case) and biochar usage (scenario I, 0% to 199.1% relative to the base case). Biochar stability could significantly influence the amount of carbon ultimately stored in the soil. The net life-cycle GHG emissions would increase dramatically (+199.1%) when 100% of biochar is sold for uses where the carbon is ultimately released into the atmosphere (scenario I).

Scenario E. Biochar stability

Based on results of stability experiments on biochar from different types of crop residues, an average 73% of the carbon in biochar is stable over long term is assumed in the base case. In order to have a better understanding of the potential impact of this factor on the net life-cycle GHG emissions from BIPP systems, a sensitivity analysis was conducted considering the change of biochar stability in the range of 60%-80%[37] (a more conservative range than that in IPCC report: 79%-91% biochar stability over a time span of 100 years[84]).

Scenario F. Transport distance

For the base case scenario, BIPP plants are assumed to be built in locations where available biomass is abundant and easily supplied with an average transport distance of 40 km (based on the BIPP demonstration plant in case study [13]). However, in extreme situations assuming use of more than 80% of the China's biomass resources in BIPP systems, the transport distance would be expanded so as to satisfy the daily feedstock requirements from a certain BIPP plant. Thus, in sensitivity analysis, biomass transport distance changing from 20 km to 100 km is considered (shown in Supplementary Table 27 and Supplementary Table 30).

Scenario G. Transport models

Based on the current status of vehicle utilization in China, the transport model of diesel vehicle is assumed in the base case life-cycle GHG emissions assessment. It is obvious that different transport models could lead to different GHG emissions. Hydrogen and electric vehicles, low-carbon transport options, may be feasible in the future, and thus have been considered in the sensitivity analysis. Supplementary Table 29 shows the energy consumption of different transport models, and the resulting net life-cycle GHG emissions from different transport models are shown in Supplementary Table 30.

Scenario H. N_2O reduction from biochar soil effect

There are significant uncertainties associated with the reduced soil N_2O emissions for various biochar application rates (e.g. biochar quantity and applied depth) and soil condition (e.g. soil type)[40]. The annual avoided soil N_2O emissions (E_N) are calculated based on Supplementary Equation (10), in which the R_N (reduction factor) has been investigated in the range of 0 - 80% in previous research[40] (60% in the base case). Based on this range, the potential N_2O reduction from biochar soil effect is thus considered in the sensitivity analysis (Supplementary Table 27).

Scenario I. Biochar usage

If biochar is not returned back to the soil (with a negative emission), it can be sold as fuels. The carbon in biochar will be released back into atmosphere (with a neutral emission). Considering the GHG emission difference between biochar sequestration in soil and use of biochar as a fuel substitute, is studied on three cases in the sensitivity analysis: 100% of biochar for sequestered in soil (base case); 60% biochar in soil sequestration + 40% sold in the industrial market; and 100% biochar selling in the industrial market (shown in Supplementary Table 27).

Supplementary Table 29: Energy consumption of different transport models[85,86]

Number	Power sources	Consumption intensity		Unit	Density	Price		Unit
		Current	From 2030		(kg L^{-1})	Current	From 2030	
1	Diesel	0.050	-	L km^{-1}	0.83	1.10	-	USD kg^{-1}
2	Gasoline	0.059	-	L km^{-1}	0.75	1.47	-	USD kg^{-1}
3	Hydrogen	-	0.007	kg km^{-1}	-	-	4.84[a]	USD kg^{-1}
4	Electricity	-	0.121	kWh km^{-1}	-	-	0.06[b]	USD kWh^{-1}

a. Average price from reference[87]

b. Is equal to the domestic electricity price

Supplementary Table 30: Net life-cycle GHG emissions of different transport distance and models

Transportation		GHG emissions (t CO_2-eq)	Net life-cycle GHG emissions (g CO_2-eq MJ^{-1})	Proportion in whole system (%)
20 km	Diesel	2.86×10^2	-53.46	4.12
	Gasoline	4.06×10^2	-52.59	5.74
	Hydrogen	2.12×10^2	-54.00	3.08
	Electricity	8.51×10^1	-54.92	1.26
40 km	Diesel	5.73×10^2	-51.38	7.92
	Gasoline	8.12×10^2	-49.65	10.86
	Hydrogen	4.24×10^2	-52.46	5.98
	Electricity	1.70×10^2	-54.30	2.49
100 km	Diesel	1.43×10^3	-45.15	17.69
	Gasoline	2.03×10^3	-40.82	23.34
	Hydrogen	1.06×10^3	-47.86	13.71
	Electricity	4.26×10^2	-52.45	6.00

Supplementary Note 24: Sensitivity analysis of modelling scenarios for China's bio-NETs deployment

Based on the results as mentioned above, for a single BIPP plant, the impact of N_2O reductions on the total life-cycle GHG emission is small (between -1.0% and 3.0%), while the variations in biochar stabilities and transport distance/models play an important role in the BIPP life cycle GHG emissions (with change rates of -19.7% to 34.6%, and -6.9% to 10.6%, respectively). Thus, the range of cumulative GHG reductions from a change in biochar stability and transport distance/models is studied further in several potential scenarios for future bio-NETs deployment in China.

The results in Supplementary Fig. 11 show that the cumulative GHG reduction by 2050 could be affected to a certain extent (-3.9% to 5.9% relative to the base case) under the scenarios 1-4 and 6-7. Furthermore, it shows that under scenario 5 (assuming deployment of BIPP only), the cumulative GHG reduction by 2050 is more sensitive (-11.3% to 17.0% relative to base case), compared with other scenarios. In this sense, BIPP coordinated with BECCS deployment will result in a lower uncertainty in future cumulative GHG reduction by 2050, compared to deployment of BIPP alone (scenario 5).

For a mid-term perspective, the initial highest biomass input in BIPP coordinated with further BECCS deployment (scenario 4) can reach up to 8337-9127 Mt CO_2-eq reduction by the end of 2050. To put this in a worldwide context, the global emissions reduction target required to meet the

Paris Agreement goal of limiting the increase in global temperatures to no more than 1.5 °C by 2050 using BECCS technology was pegged at 28-65 Gt CO_2-eq[81]. China by itself can achieve around 14-33% of the global removal goal by focusing strongly between now and 2050 on deployment of bio-NETs systems employing only agricultural, forest residues and energy plants (explained in Supplementary Note 16-20). Furthermore, the scenario using 100% of agricultural residues, energy crops and forest residues (scenario 7) could provide cumulative reduction potentials of 21018 to 22674 Mt CO_2-eq by 2050. To sum up, these options under the "moderate" or "maximum" scenarios (aggressive deployment) could contribute to 175-3599 Mt CO_2-eq of GHG reductions by 2030. These value can be translated to a reduction on carbon emissions per unit of 2005 GDP by 2%-69%, which could approach or even achieve the goals alone (i.e., 60%-65% CO_2 reduction) included in China's NDC for the Paris Agreement[88].

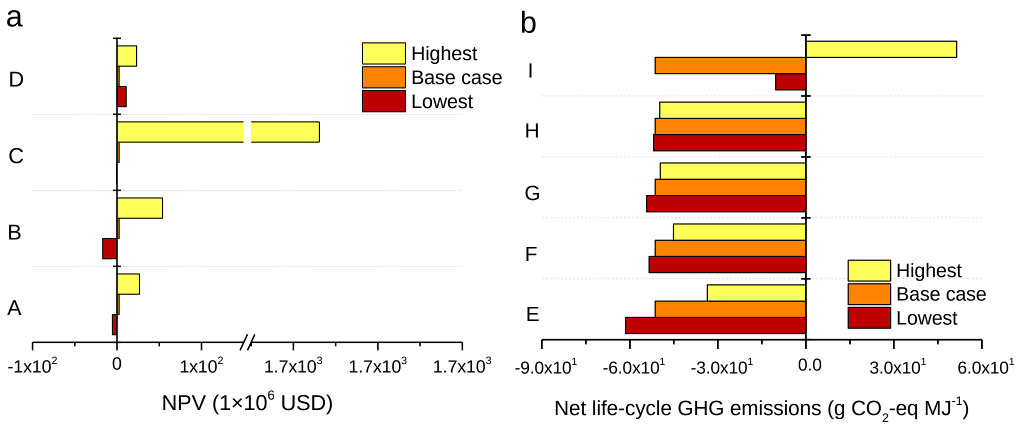

Supplementary Figure 10: The sensitivity analysis for a demonstration BIPP system. a The economic feasibility analysis for scenario A-D; **b** The life-cycle GHG emissions analysis for scenario E-I. The scenarios for highest case, base case and lowest case have been described in Supplementary Table 27.

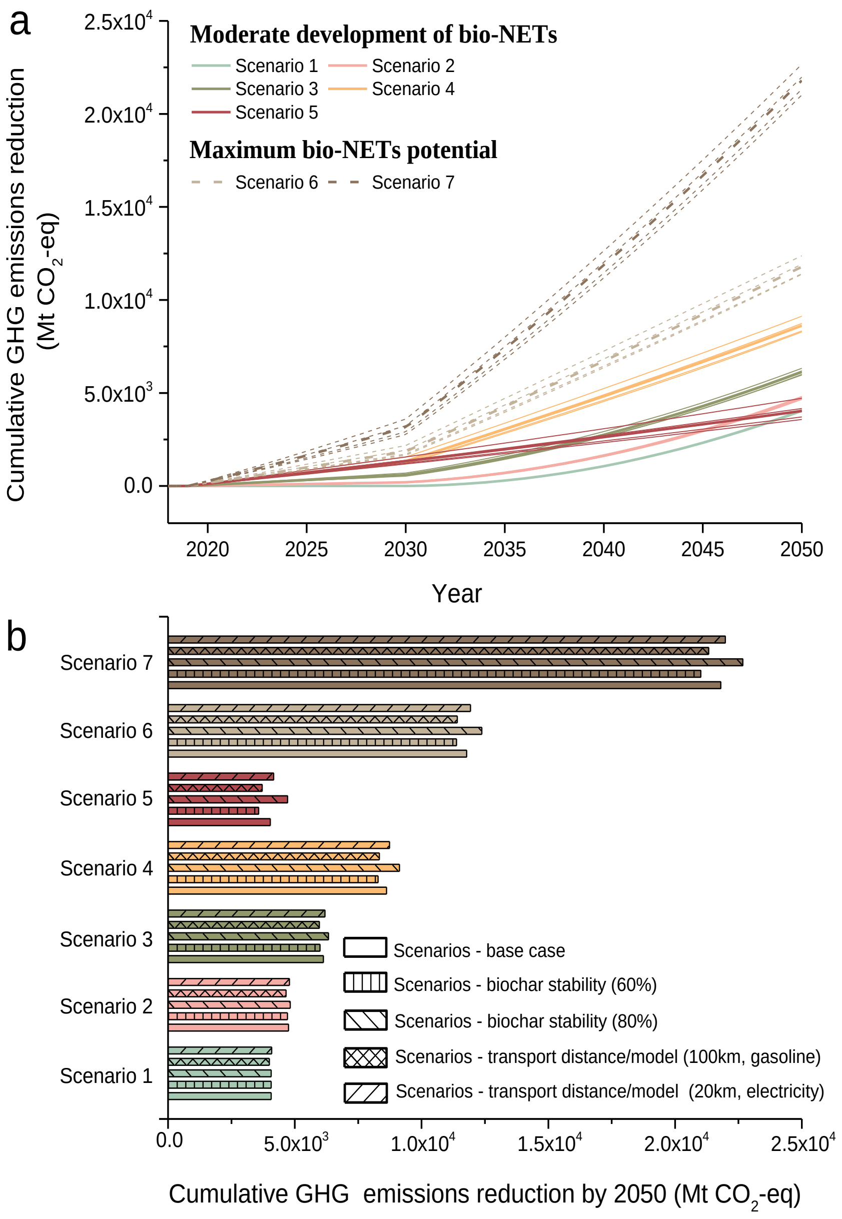

Supplementary Figure 11: The sensitivity analysis of outcomes from scenarios for China's bio-NETs deployment. **a** The cumulative GHG emissions reduction from 2020 to 2050: the thick lines indicate the results from base case for scenario 1-7, and the thin lines show the results from four different scenarios (biochar stability-60% and 80%; transport distance/model-100km, gasoline and 20km, electricity); **b** The cumulative GHG emissions reduction by 2050.

Supplementary References:

1 Debiagi, P. E. A. *et al.* Extractives Extend the Applicability of Multistep Kinetic Scheme of Biomass Pyrolysis. *Energy & Fuels* **10**, 6544-6555 (2015).

2 Shemfe, M. B., Gu, S. & Ranganathan, P. Techno-economic performance analysis of biofuel production and miniature electric power generation from biomass fast pyrolysis and bio-oil upgrading. *Fuel* **143**, 361-372 (2015).

3 Peters, J. F., Banks, S. W., Bridgwater, A. V. & Dufour, J. A kinetic reaction model for biomass pyrolysis processes in Aspen Plus. *Applied Energy* **188**, 595-603 (2017).

4 Ranzi, E. *et al.* Chemical Kinetics of Biomass Pyrolysis. *Energy & Fuels* **22**, 4292-4300 (2008).

5 Gao, Y. *et al.* Pyrolysis of rapeseed stalk: Influence of temperature on product characteristics and economic costs. *Energy* **122**, 482-491 (2017).

6 China Agricultural Yearbook. (Editorial board of China Agriculture Yearbook, Beijing, 2014).

7 Yang, H. *et al.* In-depth investigation of biomass pyrolysis based on three major components: Hemicellulose, cellulose and lignin. *Energy & Fuels* **20**, 388-393 (2011).

8 Lehmann, J. *et al.* Biochar effects on soil biota – A review. *Soil Biology and Biochemistry* **43**, 1812-1836 (2011).

9 Chen, Y. The mechanism and experimental study of biomass pyrolysis polygeneration process, (Huazhong University of Science and Technology, 2013).

10 China Statistical Yearbook. (China Statistics Press, 2016).

11 Gao, J. *et al.* An integrated assessment of the potential of agricultural and forestry residues for energy production in China. *GCB Bioenergy* **8**, 880-893, doi:10.1111/gcbb.12305 (2016).

12 Gaskin, J. *et al.* in *Amazonian Dark Earths: Wim Sombroek's Vision* 433-443 (Springer, 2009).

13 Feasibility report--Biomass pyrolysis cogeneration for demonstration project. (Hubei Electric Power Survey&Design Institute, 2013).

14 Cong, H. *et al.* Slow Pyrolysis performance and energy balance of corn stover in continuous pyrolysis-based poly-generation systems. *Energy & Fuels* **32**, 3743-3750, doi:10.1021/acs.energyfuels.7b03175 (2018).

15 Gómez, N. *et al.* Slow pyrolysis of relevant biomasses in the Mediterranean basin. Part 1. Effect of temperature on process performance on a pilot scale. *Journal of cleaner production* **120**, 181-190 (2016).

16 Mei, Y., Liu, R., Wu, W. & Zhang, L. Effect of hot vapor filter temperature on mass yield, energy balance, and properties of products of the fast pyrolysis of pine sawdust. *Energy & Fuels* **30**, 10458-10469 (2016).

17 Peters, J. F., Iribarren, D. & Dufour, J. Biomass pyrolysis for biochar or energy applications? A life cycle assessment. *Environmental Science & Technology* **49**, 5195-5202 (2015).

18 Peng, H. *et al.* Feasibility report of Jiangsu Jianhu 20MW biomass gasification gas-steam combined cycle power generation project. (Nanjing Guolian Electric Power Engineering Design Co., Ltd, 2006).

19 Zhang, X. *et al.* Application of biomass pyrolytic polygeneration by a moving bed: Characteristics of products and energy efficiency analysis. *Bioresource technology* **254**, 130-

138 (2018).

20 Xing, A., Liu, G., Wang, Y., Wei, F. & Jin, Y. The process cost, energy consumption and environmental impact analysis of biomass resources. *Chinese Journal of Process Engineering* **8**, 305-313 (2008).

21 Song, Z., Zhu, L., Lu, F. & Dai, M. Analysis of raw material collection radius of tree biomass - taking the biomass power project of jinggu county as an example. *Forestry Construction*, 66-68 (2014).

22 Bond, J. Q. *et al.* Production of renewable jet fuel range alkanes and commodity chemicals from integrated catalytic processing of biomass. *Energy & Environmental Science* **7**, 1500-1523 (2014).

23 Vlysidis, A., Binns, M., Webb, C. & Theodoropoulos, C. A techno-economic analysis of biodiesel biorefineries: Assessment of integrated designs for the co-production of fuels and chemicals. *Energy* **36**, 4671-4683 (2011).

24 Lu, X. *et al.* Gasification of coal and biomass as a net carbon-negative power source for environment-friendly electricity generation in China. *Proceedings of the National Academy of Sciences* **116**, 8206-8213 (2019).

25 Yang, Q. *et al.* Hybrid life-cycle assessment for energy consumption and greenhouse gas emissions of a typical biomass gasification power plant in China. *Journal of Cleaner Production* **205**, 661-671, doi:10.1016/j.jclepro.2018.09.041 (2018).

26 Bruun, E. W., Petersen, C. T., Hansen, E., Holm, J. K. & Hauggaard-Nielsen, H. Biochar amendment to coarse sandy subsoil improves root growth and increases water retention. *Soil use and management* **30**, 109-118 (2014).

27 Laird, D. A. *et al.* Impact of biochar amendments on the quality of a typical Midwestern agricultural soil. *Geoderma* **158**, 443-449 (2010).

28 Ulyett, J., Sakrabani, R., Kibblewhite, M. & Hann, M. Impact of biochar addition on water retention, nitrification and carbon dioxide evolution from two sandy loam soils. *European Journal of Soil Science* **65**, 96-104 (2014).

29 Amoakwah, E., Frimpong, K. A., Okae-Anti, D. & Arthur, E. Soil water retention, air flow and pore structure characteristics after corn cob biochar application to a tropical sandy loam. *Geoderma* **307**, 189-197 (2017).

30 Tang, H., Qiu, J., Van Ranst, E. & Li, C. Estimations of soil organic carbon storage in cropland of China based on DNDC model. *Geoderma* **134**, 200-206, doi:10.1016/j.geoderma.2005.10.005 (2006).

31 Wu, X. D., Guo, J. L. & Chen, G. Q. The striking amount of carbon emissions by the construction stage of coal-fired power generation system in China. *Energy Policy* **117**, 358-369, doi:10.1016/j.enpol.2018.02.043 (2018).

32 Eggleston, S., Buendia, L., Miwa, K., Ngara, T. & Tanabe, K. *2006 IPCC guidelines for national greenhouse gas inventories*. Vol. 5 (Institute for Global Environmental Strategies Hayama, Japan, 2006).

33 Al, J. E. & Paasche. *IPCC. Climate Change 2007: The Physical Science Basis. Contribution of Working Group I to the Fourth Assessment Report of the Intergovernmental Panel on Climate*

Change. Cambridge University Press: Cambridge, UK. (2007).

34 Xie, G., Wang, X. & Ren, L. China's crop residues resources evaluation. *Chinese Journal of Biotechnology* **26**, 855-863, doi:10.13345/j.cjb.2010.07.023 (2010).

35 Fan, Y. Present status of China's fertilizer industry and analysis on market demand of fertilizer. *Phosphate & Compound Fertilizer* **19**, 1-6 (2004).

36 Cross, A. & Sohi, S. P. A method for screening the relative long-term stability of biochar. *GCB Bioenergy* **5**, 215-220, doi:10.1111/gcbb.12035 (2013).

37 Spokas, K. A. Review of the stability of biochar in soils: predictability of O: C molar ratios. *Carbon Management* **1**, 289-303 (2010).

38 Boateng, A. A. *et al.* Sustainable production of bioenergy and biochar from the straw of high-biomass soybean lines via fast pyrolysis. *Environmental Progress & Sustainable Energy* **29**, 175-183 (2010).

39 Troy, S. M., Lawlor, P. G., O'Flynn, C. J. & Healy, M. G. Impact of biochar addition to soil on greenhouse gas emissions following pig manure application. *Soil Biology and Biochemistry* **60**, 173-181 (2013).

40 Woolf, D., Amonette, J. E., Street-Perrott, F. A., Lehmann, J. & Joseph, S. Sustainable biochar to mitigate global climate change. *Nature Communications* **1**, 56, doi:10.1038/ncomms1053 (2010).

41 Straw Comprehensive Utilization Technology Catalogue. (National Development and Reform Commission, 2014).

42 Wang, H. *et al.* Chinese crop straw resource and its utilization status. *Science & Technology Review* **35**, 81-88, doi:10.3981/j.issn.10007857.2017.21.010 (2017).

43 Ni, H. *et al.* Emission characteristics of carbonaceous particles and trace gases from open burning of crop residues in China. *Atmospheric Environment* **123**, 399-406, doi:10.1016/j.atmosenv.2015.05.007 (2015).

44 Sillapapiromsuk, S., Chantara, S., Tengjaroenkul, U., Prasitwattanaseree, S. & Prapamontol, T. Determination of PM10 and its ion composition emitted from biomass burning in the chamber for estimation of open burning emissions. *Chemosphere* **93**, 1912-1919, doi:10.1016/j.chemosphere.2013.06.071 (2013).

45 Fu, X. *et al.* Emission inventory of primary pollutants and chemical speciation in 2010 for the Yangtze River Delta region, China. *Atmospheric Environment* **70**, 39-50, doi:https://doi.org/10.1016/j.atmosenv.2012.12.034 (2013).

46 Zhao, Y., Wang, S., Nielsen, C. P., Li, X. & Hao, J. Establishment of a database of emission factors for atmospheric pollutants from Chinese coal-fired power plants. *Atmospheric Environment* **44**, 1515-1523, doi:10.1016/j.atmosenv.2010.01.017 (2010).

47 Liu, X. & Yuan, Z. Life cycle environmental performance of by-product coke production in China. *Journal of Cleaner Production* **112**, 1292-1301, doi:10.1016/j.jclepro.2014.12.102 (2016).

48 Pachauri, R. K. & Reisinger, A. IPCC fourth assessment report. *IPCC, Geneva*, 2007 (2007).

49 Kemper, J. Biomass and carbon dioxide capture and storage: A review. *International Journal of Greenhouse Gas Control* **40**, 401-430 (2015).

50 Coninck, H. d. *et al.* Strengthening and implementing the global response. *An IPCC special report on the impacts of global warming of 1.5 °C above pre-industrial levels and related global greenhouse gas emission pathways, in the context of strengthening the global response to the threat of climate change, sustainable development, and efforts to eradicate poverty* (2018).

51 Woolf, D., Lehmann, J. & Lee, D. R. Optimal bioenergy power generation for climate change mitigation with or without carbon sequestration. *Nature Communications* **7**, 13160, doi:10.1038/ncomms13160 (2016).

52 Heuberger, C., Staffell, I., Shah, N. & Dowell, N. M. Quantifying the value of CCS for the future electricity system. *Energy & Environmental Science* **9** (2016).

53 Fridahl, M. Socio-political prioritization of bioenergy with carbon capture and storage. *Energy Policy* **104**, 89-99 (2017).

54 Fuss, S. *et al.* Negative emissions—Part 2: Costs, potentials and side effects. *Environmental Research Letters* **13**, 063002, doi:10.1088/1748-9326/aabf9f (2018).

55 Pachauri, R. K. *et al. Climate change 2014: synthesis report. Contribution of Working Groups I, II and III to the fifth assessment report of the Intergovernmental Panel on Climate Change*. (IPCC, 2014).

56 Tanaka, K. & O'Neill, B. C. The Paris Agreement zero-emissions goal is not always consistent with the 1.5 °C and 2 °C temperature targets. *Nature Climate Change* **8**, 319-324, doi:10.1038/s41558-018-0097-x (2018).

57 Kato, E. & Yamagata, Y. BECCS capability of dedicated bioenergy crops under a future land-use scenario targeting net negative carbon emissions. *Earth's Future* **2**, 421-439, doi:10.1002/2014ef000249 (2014).

58 Tai, A. P. K., Martin, M. V. & Heald, C. L. Threat to future global food security from climate change and ozone air pollution. *Nature Climate Change* **4**, 817-821, doi:10.1038/nclimate2317 (2014).

59 Mueller, N. D. *et al.* Closing yield gaps through nutrient and water management. *Nature* **494**, 390-390 (2013).

60 Zhuo, L., Mekonnen, M. M. & Hoekstra, A. Y. Consumptive water footprint and virtual water trade scenarios for China—With a focus on crop production, consumption and trade. *Environment international* **94**, 211-223 (2016).

61 China Renewable Energy Industry Development Report. (China National Renewable Energy Centre, 2015).

62 China Wind, Solar and Bioenergy Roadmap 2050-Sino-Danish Renewable Energy Development Programme. (China National Renewable Energy Centre, 2014).

63 Tran, C. *et al.* Effect of different sources of roughages [wheat straw, rice straw and corn stover] on the efficiency of feed utilization in fattened Frisian calves. *Agricultural Research Review* **324**, 787–790 (1975).

64 Yang, Q. & Chen, G. Q. Nonrenewable energy cost of corn-ethanol in China. *Energy Policy* **41**, 340-347, doi:10.1016/j.enpol.2011.10.055 (2012).

65 Basu, P. *Biomass gasification and pyrolysis: practical design and theory*. (Academic press,

2010).
66 Study on Energy Emission Scenarios in China's Global 1.5°C Target. (National Development and Reform Commission of China, 2018).
67 Buss, W., Jansson, S., Wurzer, C. & Masˇek, O. e. Synergies between BECCS and Biochar—maximizing carbon sequestration potential by recycling wood ash. *ACS Sustainable Chemistry & Engineering* **7**, 4204-4209 (2019).
68 Wu, Y., Xu, Z. & Li, Z. Lifecycle analysis of coal-fired power plants with CCS in China. *Energy Procedia* **63**, 7444-7451 (2014).
69 Spath, P. L. & Mann, M. K. Biomass Power and Conventional Fossil Systems with and without CO_2 Sequestration--Comparing the Energy Balance, Greenhouse Gas Emissions and Economics. *National Renewable Energy Laboratory (NREL)* (2004).
70 Chang, Y., Huang, R., Ries, R. J. & Masanet, E. Life-cycle comparison of greenhouse gas emissions and water consumption for coal and shale gas fired power generation in China. *Energy* **86**, 335-343 (2015).
71 Lacy, R. *et al.* Life-cycle GHG assessment of carbon capture, use and geological storage (CCUS) for linked primary energy and electricity production. *International Journal of Greenhouse Gas Control* **42**, 165-174, doi:10.1016/j.ijggc.2015.07.017 (2015).
72 Schakel, W., Meerman, H., Talaei, A., Ramírez, A. & Faaij, A. Comparative life cycle assessment of biomass co-firing plants with carbon capture and storage. *Applied Energy* **131**, 441-467, doi:10.1016/j.apenergy.2014.06.045 (2014).
73 Koornneef, J., van Keulen, T., Faaij, A. & Turkenburg, W. Life cycle assessment of a pulverized coal power plant with post-combustion capture, transport and storage of CO_2. *International Journal of Greenhouse Gas Control* **2**, 448-467, doi:10.1016/j.ijggc.2008.06.008 (2008).
74 Xue, S., Lewandowski, I., Wang, X. & Yi, Z. Assessment of the production potentials of Miscanthus on marginal land in China. *Renewable and Sustainable Energy Reviews* **54**, 932-943, doi:10.1016/j.rser.2015.10.040 (2016).
75 Zhuang, D., Jiang, D., Liu, L. & Huang, Y. Assessment of bioenergy potential on marginal land in China. *Renewable and Sustainable Energy Reviews* **15**, 1050-1056 (2011).
76 Parajuli, R., Sperling, K. & Dalgaard, T. Environmental performance of Miscanthus as a fuel alternative for district heat production. *Biomass and bioenergy* **72**, 104-116 (2015).
77 Wang, H., Zuo, X., Wang, D. & Bi, Y. The estimation of forest residue resources in China. *Journal of Central South University of Forestry & Technology* **37**, 29-38 (2015).
78 China Forestry Statistical Yearbook 2016. (The state forestry administration of the People's Republic of China, Beijing, 2016).
79 China Forest Resources Report 2009-2013. (The state forestry administration of the People's Republic of China Beijing, 2014).
80 The Future of Food and Agriculture Trends and Challenges. (Food and Agriculture Organization of the United Nations (FAO), Rome, 2017).
81 Rogelj, J. *et al.* Mitigation pathways compatible with 1.5° C in the context of sustainable development. *An IPCC special report on the impacts of global warming of 1.5°C above pre-industrial levels and related global greenhouse gas emission pathways, in the context of*

strengthening the global response to the threat of climate change, sustainable development, and efforts to eradicate poverty (2018).

82 Balat, M., Balat, M., Kırtay, E. & Balat, H. Main routes for the thermo-conversion of biomass into fuels and chemicals. Part 1: Pyrolysis systems. *Energy Conversion and Management* **50**, 3147-3157, doi:10.1016/j.enconman.2009.08.014 (2009).

83 Sönnichsen, N. *Average annual Brent crude oil price from 1976 to 2020*, <https://www.statista.com/statistics/262860/uk-brent-crude-oil-price-changes-since-1976/> (2020).

84 Eduardo, C. *et al.* Refinement to the 2006 IPCC Guidelines for National Greenhouse Gas Inventories. *The Intergovernmental Panel on Climate Change* (2019).

85 Miotti, M., Hofer, J. & Bauer, C. Integrated environmental and economic assessment of current and future fuel cell vehicles. *The International Journal of Life Cycle Assessment* **22**, 94-110 (2017).

86 China Energy Statistical Yearbook. (China Statistics Press, 2008).

87 Jian, L. & Caifu, Z. Hydrogen energy development status and prospects in China. *Energy of China* **41**, 32-36, doi:10.3969/j.issn.1003-2355.2019.02.007 (2019).

88 China's National Determined Contribution submitted to the UN Framework Convention on Climate Change, <http://www.gov.cn/xinwen/2015-06/30/content_2887330.htm. > (2015).
