# Supplementary material for: Prospective contributions of biomass pyrolysis to China’s 2050 carbon reduction and renewable energy goals
Source: Nat Commun. 2021 Mar 16;12:1698. doi: 10.1038/s41467-021-21868-z (PMC7966788; doi:10.1038/s41467-021-21868-z)
Supplement: Supplementary file 3 — Reporting Summary [file 41467_2021_21868_MOESM3_ESM.pdf]

nature research

Corresponding author(s): Michael B. McElroy

Last updated by author(s): Feb 4, 2021

Reporting Summary

Nature Research wishes to improve the reproducibility of the work that we publish. This form provides structure for consistency and transparency in reporting. For further information on Nature Research policies, see our Editorial Policies and the Editorial Policy Checklist.

Statistics

For all statistical analyses, confirm that the following items are present in the figure legend, table legend, main text, or Methods section.

n/a	Confirmed	
☐	☒	The exact sample size (*n*) for each experimental group/condition, given as a discrete number and unit of measurement
☒	☐	A statement on whether measurements were taken from distinct samples or whether the same sample was measured repeatedly
☒	☐	The statistical test(s) used AND whether they are one- or two-sided<br>*Only common tests should be described solely by name; describe more complex techniques in the Methods section.*
☐	☒	A description of all covariates tested
☐	☒	A description of any assumptions or corrections, such as tests of normality and adjustment for multiple comparisons
☐	☒	A full description of the statistical parameters including central tendency (e.g. means) or other basic estimates (e.g. regression coefficient) AND variation (e.g. standard deviation) or associated estimates of uncertainty (e.g. confidence intervals)
☒	☐	For null hypothesis testing, the test statistic (e.g. *F*, *t*, *r*) with confidence intervals, effect sizes, degrees of freedom and *P* value noted<br>*Give P values as exact values whenever suitable.*
☒	☐	For Bayesian analysis, information on the choice of priors and Markov chain Monte Carlo settings
☒	☐	For hierarchical and complex designs, identification of the appropriate level for tests and full reporting of outcomes
☒	☐	Estimates of effect sizes (e.g. Cohen's *d*, Pearson's *r*), indicating how they were calculated

Our web collection on statistics for biologists contains articles on many of the points above.

Software and code

Policy information about availability of computer code

Data collection	No software was used.
Data analysis	Aspen Plus v11 was used for model construction and system analysis. Matlab R2018a was used for economic and environmental analysis. ArcGIS 10.2 was used for conducting spatial analysis.

For manuscripts utilizing custom algorithms or software that are central to the research but not yet described in published literature, software must be made available to editors and reviewers. We strongly encourage code deposition in a community repository (e.g. GitHub). See the Nature Research guidelines for submitting code & software for further information.

Data

Policy information about availability of data

All manuscripts must include a data availability statement. This statement should provide the following information, where applicable:

- Accession codes, unique identifiers, or web links for publicly available datasets
- A list of figures that have associated raw data
- A description of any restrictions on data availability

All data to generate Figures 1-6 are available included in this published article (in the Supplementary Dataset file). The datasets to generate the maps are generated from reference (Applied energy 184, 742-750 (2016)) and calculations (shown in Supplementary Dataset file). The collected data, such as data from BIPP demonstration projects, in this study are available from Qing Yang and Hewen Zhou upon any reasonable requests.

Field-specific reporting

Please select the one below that is the best fit for your research. If you are not sure, read the appropriate sections before making your selection.

- [ ] Life sciences
- [ ] Behavioural & social sciences
- [x] Ecological, evolutionary & environmental sciences

For a reference copy of the document with all sections, see nature.com/documents/nr-reporting-summary-flat.pdf

Ecological, evolutionary & environmental sciences study design

All studies must disclose on these points even when the disclosure is negative.

Study description	Assessment of Biomass Intermediate Pyrolysis Poly-generation (BIPP) systems in China and exploration of carbon negative potential of China's BIPP deployment in near future.
Research sample	This is not relevant to our study
Sampling strategy	This is not relevant to our study
Data collection	Hewen Zhou has collected all the data. Some data is important, measured and collected from BIPP demonstration project in China, relevant experiments (Energy 122, 482-491 (2017)) and real market (website of Alibaba:www.1688.com); other existing and accessible datasets are from public reports (e.g., China Statistical Yearbook) and literature.
Timing and spatial scale	This is not relevant to our study
Data exclusions	No data were excluded.
Reproducibility	To ensure reproducibility, the experiments for biochar stability listed in Supplementary Table 16 has been repeated three times. Model simulation, economic and environmental analysis were described in supplementary Information and related data are provided in Supplementary dataset file, everything is reproducible.
Randomization	No relevant to our study, because no comparative experiment was involved.
Blinding	No relevant to our study, because no data collection nor comparative experiment was involved.

Did the study involve field work? [ ] Yes [x] No

Reporting for specific materials, systems and methods

We require information from authors about some types of materials, experimental systems and methods used in many studies. Here, indicate whether each material, system or method listed is relevant to your study. If you are not sure if a list item applies to your research, read the appropriate section before selecting a response.

Materials & experimental systems

n/a	Involved in the study	
[x]	[ ]	Antibodies
[x]	[ ]	Eukaryotic cell lines
[x]	[ ]	Palaeontology and archaeology
[x]	[ ]	Animals and other organisms
[x]	[ ]	Human research participants
[x]	[ ]	Clinical data
[x]	[ ]	Dual use research of concern

Methods

n/a	Involved in the study	
[x]	[ ]	ChIP-seq
[x]	[ ]	Flow cytometry
[x]	[ ]	MRI-based neuroimaging
